# Antidiabetic Effect of Galantamine: Novel Effect for a Known Centrally Acting Drug

**DOI:** 10.1371/journal.pone.0134648

**Published:** 2015-08-11

**Authors:** Mennatallah A. Ali, Hanan S. El-Abhar, Maher A. Kamel, Ahmed S. Attia

**Affiliations:** 1 Department of Pharmacology & Toxicology, Faculty of Pharmacy and Drug Manufacturing, Pharos University, Alexandria, Egypt; 2 Department of Pharmacology & Toxicology, Faculty of Pharmacy, Cairo University, Cairo, Egypt; 3 Department of Biochemistry, Medical Research Institute, Alexandria University, Alexandria, Egypt; 4 Department of Microbiology and Immunology, Faculty of Pharmacy, Cairo University, Cairo, Egypt; Emory University, UNITED STATES

## Abstract

The cholinergic anti-inflammatory pathway is one of the putative biochemical pathways that link diabetes with Alzheimer disease. Hence, we aimed to verify the potential antidiabetic effect of galantamine, unveil the possible mechanisms and evaluate its interaction with vildagliptin. The n5-STZ rat model was adopted and the diabetic animals were treated with galantamine and/or vildagliptin for 4 weeks. Galantamine lowered the n5-STZ-induced elevation in body weight, food/water intake, serum levels of glucose, fructosamine, and ALT/AST, as well as AChE in the tested organs. Moreover, it modulated successfully the lipid profile assessed in serum, liver, and muscle, and increased serum insulin level, as well as % β-cell function, in a pattern similar to that of vildagliptin. Additionally, galantamine confirmed its antioxidant (Nrf2, TAC, MDA), anti-inflammatory (NF-κB, TNF-α, visfatin, adiponectin) and anti-apoptotic (caspase-3, cytochrome c) capabilities by altering the n5-STZ effect on all the aforementioned parameters. On the molecular level, galantamine/vildagliptin have improved the insulin (p-insulin receptor, p-Akt, GLUT4/GLUT2) and Wnt/β-catenin (p-GSK-3β, β-catenin) signaling pathways. On almost all parameters, the galantamine effects surpassed that of vildagliptin, while the combination regimen showed the best effects. The present results clearly proved that galantamine modulated glucose/lipid profile possibly through its anti-oxidant, -apoptotic, -inflammatory and -cholinesterase properties. These effects could be attributed partly to the enhancement of insulin and Wnt/β-catenin signaling pathways. Galantamine can be strongly considered as a potential antidiabetic agent and as an add-on therapy with other oral antidiabetics.

## Introduction

The immuno-modulatory/anti-inflammatory function of the cholinergic pathway plays a role in chronic inflammatory disorders, including diabetes and Alzheimer disease. Recently, the coexistence of the two ailments is documented by ample of evidence, where they share some common pathological events, among which are increased acetylcholine esterase (AChE) activity, oxidative stress, apoptosis and the release of inflammatory mediators [1].

The natural course of type 2 diabetes mellitus (T2DM) is characterized by chronic hyperglycemia, insulin abnormalities, and progressive β-cell dysfunction. Despite these metabolic defects are partly ascribed to increased oxidative stress, inflammation and apoptosis [2], yet the specific underlying determinants remain uncertain.

Nowadays, the effect of diabetes on the central nervous system (CNS) is gaining importance [3]; the brain cholinergic signaling is implicated in the regulation of cytokines release [4], hepatic glucose/ glycogen production *via* efferent vagus nerve cholinergic output [5] and acetylcholine (ACh) facilitates the release of insulin in a glucose-dependent manner. It is likely that these vagus nerve-mediated cholinergic mechanisms, involved in glucose homeostasis, become suppressed or dysfunctional in obesity and insulin resistance (I/R), as indicated by the autonomic imbalance and lower vagal tone in obese individuals [5]. Moreover, AChE activity was proven to increase in diabetic models, which indicates alteration in the cholinergic neurotransmission with the consequent cognitive impairments observed in the diabetic state [3]. Hence, it is now plausible that targeting AChE, to increase the cholinergic pathway activity, could be beneficial in the management of T2DM.

Apart from the insulin signaling pathway, other pathways are involved in this ailment including the canonical Wnt (wingless-type MMTV integration site family) signaling pathway, which plays a role in the healing of diabetic foot ulcer [6], and regeneration of insulin-producing pancreatic cells [7]. Moreover, the Wnt downstream effector, β-catenin, participates in the pancreatic development and is implicated in the regulation of metabolism and energy homeostasis [8].

Galantamine, a drug approved clinically in the treatment of Alzheimer disease, is a tertiary alkaloid that acts centrally as an AChE inhibitor, an allosteric potentiating ligand of the neuronal cholinergic nicotinic receptors [9], and possesses significant anti-inflammatory [5] and antioxidant [10] effects. In addition, it was reported that stimulation of the nicotinic receptor activates PI3K/Akt pathway, which plays a key role in the glucose homeostasis [11]. Furthermore, in 2009, Wills [12] has reported, in his patency, that AChE inhibitors, *viz*., donepezil, galantamine and rivastigmine, lower glycated hemoglobin in diabetic patients, which necessitates the adjustment of the antidiabetic therapy used.

Based on the previous data, our goal is to evaluate the possible antidiabetic effect/mechanisms of galantamine, using a T2DM animal model, and targeting its modulatory effect on glucose homeostasis, lipid profile, I/R, apoptosis and oxidative stress. The study tackled also the possible participation of the Wnt/ β-catenin and insulin signaling pathways in the galantamine effect.

Since new approaches for controlling T2DM are to target different pathways implicated in its pathogenesis, the study entailed evaluating the potential beneficial effect of galantamine as an add-on drug to vildagliptin, the standard drug used in this study.

## Material and Methods

### Induction of the Neonatal Streptozotocin (n5-STZ) Rat Model

In the present study, 5 days old male Wistar albino pups (Pharos University, Alexandria, Egypt) were divided into 2 groups, the normal control group (n = 10), in which animals received citrate buffer only and the diabetic group (n = 80), where pups were injected with freshly prepared STZ (90 mg/kg, i.p; Sigma, St. Louis, MO, USA,) in citrate buffer. On day 21, the pups were weaned and the normal control animals were maintained on a standard rat chow diet, while those in the second group were kept on cafeteria diet (a mixture of chocolate, cookies and standard rat chow, containing approximately 57.7% carbohydrate, 19.5% protein and 22.8% fat by calories) to induce the n5-STZ type 2 model of diabetes [13]. Twelve weeks post induction; rats with fasting blood glucose level ≥160 mg/dl were considered diabetic and were included in the study. All rats had free access to water and the corresponding food with 12:12 hr light/dark cycle, normal humidity, and good sanitary constant environmental conditions prior to experimentation and thereafter.

### Ethics Statement

The current protocol was approved by the “Research Ethical Committee” of the Faculty of Pharmacy, Cairo University, Cairo, Egypt (PT: 576) and all procedures were performed in strict accordance to the recommendations of the “National Research Council’s Guide for the Care and Use of Laboratory Animals of the National Institutes of Health” [14]. All procedures adhere to the ARRIVE Guidelines for reporting animal research. A completed ARRIVE guidelines checklist is included in S1 Checklist. All efforts were made to minimize the suffering of rats during the experimental period.

### Experimental Design

Besides the normal control group, the diabetic rats were randomly allocated into eight subgroups (n = 10/group) and were kept on the same cafeteria diet until the end of the experimental period. Of which, group II represented the untreated diabetic animals (receiving the vehicle), while those in groups III, IV and V were treated with galantamine (Reminyl solution, Janssen Pharmaceutics N.V., Belgium) in a dose of 2.5 [15], 5 [16] and 10 mg/kg/day [17], respectively. The following three groups *viz*., VI, VII, VIII, were administered vildagliptin (Novartis Pharma AG, Switzerland) in a dose of 3, 10 [18] and 30 mg/kg [19], respectively. These doses were chosen to test the dose dependent effects of galantamine and vildagliptin. Rats in the last diabetic group were treated with a combination of galantamine (5 mg/kg) and vildagliptin (30 mg/kg) based on the dose dependent activity. Treatments were gavaged orally at 09:00 am for 4 weeks to mimic the route of administration of these drugs in humans. The changes in body weight, as well as food (gm) and water (ml) intake were monitored during the experimental period.

### Oral Glucose Tolerance Test (OGTT)

OGTT was carried out for overnight fasted rats and the blood droplets were collected from the tail vein (baseline); afterwards, glucose (2.5 g/kg) was gavaged orally and blood samples were collected at 30, 60, 90, and 120 min. later. AUC was calculated for blood glucose levels during the OGTT according to the following equation:

$$\text{AUC = 0.25 }\times \;(\text{fasting value})\text{ + 0.5 }\times \;(\frac{1}{2}\text{ h value})\text{ + 0.75 }\times \;(\text{1 h value})\text{ + 0.5 }\times \;(\text{2 h value})\;$$

[20]

### Determination of Serum Biomarkers

At the end of the treatment period, the overnight fasted animals were deeply anaesthetized using diethyl ether and the blood was collected *via* cardiac puncture and centrifuged (800×g, 4°C, 20 min) to separate the sera, which were used to assess the following parameters. Fasting serum glucose, AST and ALT were assessed using Randox colorimetric reagent kits (Antrim, UK); fructosamine was determined using a commercial test kit (Fructosamina, Wiener, Rosario, Argentina) and insulin by an ELISA kit (Abnova, Jhongli, Taiwan). For lipid profile, the serum contents of triglycerides (TGs)/total cholesterol (TC) were determined using Boehringer Mannheim colorimetric kits (Mannheim, Germany), whereas Abcam colorimetric kit (Cambridge, UK) was used to assess free fatty acids (FFAs). HDL-C was determined according to the method described by Lopes-Virella *et al*. [21]; one aliquot of the serum was mixed with the precipitating reagent phosphotungstic acid and magnesium chloride then the cholesterol content was evaluated in the clear supernatant using the Boehringer Mannheim kit (Mannheim, Germany). Finally, LDL-C was calculated according to the Friedewald equation:

$$\text{LDL-C = TC - }(\text{HDL-C + }\frac{1}{5}\text{ TGs})$$

[22]

The homeostasis model assessment index for insulin resistance (HOMA-IR) was determined using the following formula:

$$\text{HOMA-IR = }\frac{\text{[fasting glucose }(\text{mg/dl) }\times \text{ fasting insulin }(\text{μU/ml)]}}{\text{405}}$$

[23], while the formula used for calculating the homeostatic model assessment for β-cell function (HOMA-%β) was as follows:

$$\text{HOMA-\%}\beta \text{ = }\frac{\text{20 }\times \text{ fasting insulin }\left(\mu \text{U/ml}\right)}{\text{fasting glucose }\left(\text{mg/dl}\right)\;-\text{ 63}}$$

[23].

The content of TNF-α, visfatin, and adiponectin were analyzed using commercially available ELISA kits (Biosource International, Camarillo, USA, Uscn Life Science Inc., Wuhan, China, and Abcam, Cambridge, UK, respectively).

### Determination of Tissue Parameters

Immediately after blood collection, animals were euthanized using deep diethyl ether anesthesia and their livers, muscles, and brains were excised, homogenized, divided into aliquots and preserved at -80°C until assay.

### Assessment of Tissue Lipid Profile

Hepatic/muscle lipids were extracted according to the method modified by Bligh and Dyer [24], where the chloroformic layer, containing all lipids, was utilized to assay TGs and TC, as mentioned before and the tissue contents of FFAs (μM/ gm tissue) were measured using the Abcam kit (Cambridge, UK).

### Assessment of AChE Activity

The AChE enzyme activity (mU/ mg protein) was evaluated in brain, liver, and muscle by measuring the DTNB (5,5’-Dithiobis-2-nitrobenzoic acid) adduct using a colorimetric assay kit (Abcam, Cambridge, UK).

### Assessment of Antioxidant Parameters

The Nrf2 transcription activity was determined to assess the antioxidant properties of the drugs, where the ‘‘master regulator” of the antioxidant response, Nrf2, modulates the gene expression of antioxidant enzymes. The Nrf2 activation and the antioxidant response element-binding efficacy were evaluated in the liver and muscle nuclear extracts using a Trans AM Nrf2 kit (Active Motif, Carlsbad, CA, USA). Aliquots of 10μg protein of the nuclear extract were incubated with immobilized oligonucleotides containing the antioxidant response element consensus binding site (5′-GTCACAGTACTCAGCAGAATCTG-3′) and the active form of Nrf2, which binds to the oligonucleotides, was detected using an anti-Nrf2 primary antibody after treating with horseradish peroxidase-conjugated secondary antibody. Quantitative analysis of Nrf2 (μg/ mg nuclear protein) was performed by measuring the chromogen formed as a result of specific activity of the transcription factor in the nuclear extracts using a plate reader at 450 nm [25].

The hepatic and muscular total antioxidant capacity (TAC) was also measured using Antioxidant Assay kit (Cayman Chemical Co., MI, USA). The assay relies on the ability of the sample antioxidants to inhibit the oxidation of ABTS (2,2’-Azino-di-[3-ethylbenzthiazoline sulphonate]) to ABTS^•+^ by metmyoglobin. The amount of ABTS^•+^ produced was measured by reading the absorbance at 405 nm. Under the reaction condition used, the antioxidants in the sample caused suppression of the absorbance to a degree that is proportional to their concentration. The capacity of the antioxidants in the sample to prevent ABTS oxidation was compared with that of Trolox, a water-soluble tocopherol analogue, and is quantified as Trolox equivalents (mM). Additionally, the method of Mihara and Uchiyama [26] was adopted for the assessment of malondialdehyde (MDA; nmol/gm tissue).

### Assessment of Apoptotic Parameters

Apoptosis was evaluated by measuring caspase-3 (ng/ mg protein) using caspase-3 ELISA kit (Uscn Life Science Inc., Wuhan, China) and cytochrome c (μg/ mg protein), which was determined by cytochrome c ELISA kit (Abcam, Cambridge, UK).

### Assessment of NF-κB and Signaling Pathways of Wnt/β-Catenin and Insulin

A commercially available ELISA kit for NF-κB p65 and phosphorylated insulin receptor ([pYpY1162/1163] ELISA phosphoELISA Kit) were purchased from Invitrogen (Camarillo, CA, USA). A commercially available Upstate colorimetric Signal Transduction Assay Reaction (STAR) ELISA kit was used to measure p-Akt (Threonine 308) in cellular lysate (Millipore, Billerica, MA, USA). The measurement of GLUT2 in liver and GLUT4 in muscle was performed by ELISA assay (Uscn Life Science Inc., Wuhan, China). Phosphorylated glycogen synthase kinase-3β (p-GSK-3β) at serine 9 and β-catenin were determined using the corresponding ELISA kit (Enzo Life Sciences, Plymouth Meeting, PA, USA). The protein concentrations were determined using the modified Lowry method [27].

### Muscle *GLUT4* Expression Using RT-PCR Analysis

For semi-quantitative determination of the gene expression of *GLUT4*, the One-step RT-PCR Master Mix Gold Beads kit was used. The following primer set was used, forward 5ʹ-GCAGCGAGTGACTG GAACA-3ʹ, reverse 5ʹ-CCAGCCACGTTGCATTGTAG-3ʹ. To standardize the amount of mRNA in each sample, RT-PCR of glyceraldehyde-3-phosphate dehydrogenase (*GAPDH*) was performed in parallel, using the following primer set: forward 5ʹ-AATGTGTCCGTCGTGGATCTGA-3ʹand reverse 5ʹGATGCCTGCTTCACCACCTTCT-3ʹ [28]. The cDNA synthesis was performed at 42°C for 60 min. then 94°C for 5 min. for reverse transcriptase inactivation. The resulting cDNA was amplified by PCR of 30 cycles of denaturation step; 94°C for 45 seconds, primer annealing; 56°C for 45 seconds, extension step; 72°C for 1 minute and final extension step 72°C for 10 minutes. At the end of the program, the RT-PCR product was run on 1.5% agarose and stained with ethidium bromide. The bands were visualized (UV transluminator), scanned and analyzed using UVP DOC-ITLSTM image acquisition and analysis software (Ultra-Violet product, Ltd. Cambridge, UK) that analyzes the relative band density to the *GAPDH* band.

### Statistical Analysis

Values are expressed as mean ± S.E.M of 10 animals. The GraphPad Prism v5.0 (GraphPad Prism Inc., La Jolla, CA, USA) was used to analyze and present all the data. Multiple comparisons were performed using one-way ANOVA, followed by Tukey post-hoc test. To test for an interaction between individual treatments when given in combination, a factorial design test is used. The correlation coefficient (r) between HOMA-IR with AChE enzyme activity in brain, liver, and muscle, as well as with Nrf2 transcription activity, NF-κB, p-GSK-3β, and β-catenin in liver and muscle was carried out in the untreated and treated diabetic animals using Pearson correlation coefficient; *P*< 0.05 was considered as the significance limit for all comparisons.

## Results

### Body Weight, Food Intake, and Water Intake

The n5-STZ model increased food intake that was reflected on the animals’ weight gain as compared to the normal ones (Table 1). Galantamine treated groups, but not vildagliptin, lowered both parameters, as compared to the untreated diabetic group (*P*< 0.001); this decreasing effect was best noticed in the combination regimen treated group. Diabetic rats consumed water more than the normal animals and all treatment regimens minimized water intake, an effect that could be related to the diabetic state.

**Table 1:** Effect of different treatments on body weight, as well as food and water intake in n-STZ diabetic rats.

Experimental Groups	Body weight (gm)	Food intake (gm/rat/day)	Water intake (ml/rat/day)
Normal control	225.8± 5.13	19.68 ± 0.93	60.23 ± 2.08
Diabetic control	320.6 ± 8.34*	41.24 ± 1.33*	147.51 ± 3.62*
Galan 2.5	281.5 ± 6.89^#^	34.16 ± 0.90*^#ϕ^	113.21 ± 2.79*^#ηϕ^
Galan 5	272.2 ± 5.16^#^	30.21 ± 1.10*^#^	95.84 ± 3.42*^#^
Galan 10	275.2 ± 6.60^#^	27.45 ± 0.77*^#^	86.67 ± 2.39*^#^
Vilda 3	304.6 ± 7.51*	39.57 ± 1.55*	126.16 ± 3.46*^#Ψ^
Vilda 10	308.6 ± 7.94*	40.26 ± 1.58*	110.25 ± 3.77*^#Ψ^
Vilda 30	306.1 ± 7.50*	37.89 ± 1.21*	90.43 ± 2.76*^#^
Galan 5+Vilda 30	268.4 ± 6.31^#Ψa^	23.13 ± 0.88^# ηΨb^	77.92 ± 2.80*^#ηΨ^

Galantamine (Galan 2.5, 5 & 10 mg/kg), vildagliptin (Vilda 3, 10 & 30 mg/kg) and their combination (Galan 5 & Vilda 30) were gavaged orally for four weeks. Values are means of 10 rats ± S.E.M as compared with normal control (*), diabetic control (#), Galan5 (η), Galan10 (ϕ) and Vild30 (Ψ)-treated groups (one-way ANOVA followed by Tukey post hoc test) at *P* < 0.05. (a) Additive and (b) synergistic interactions when Galan5 and Vilda30 were combined using Factorial Design.

### Oral Glucose Tolerance Test (OGTT)

**Fig 1:**
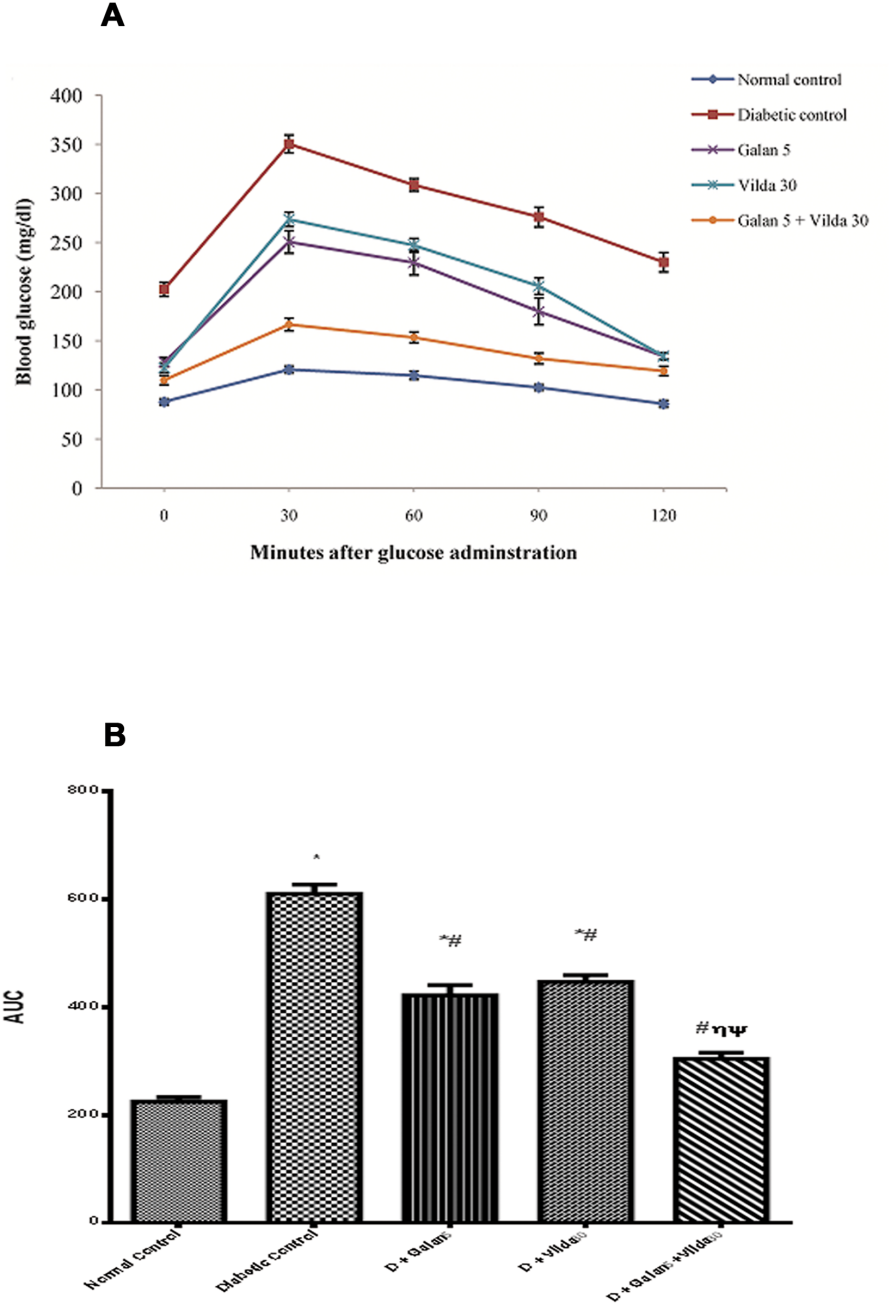
Effect of different treatments on oral glucose tolerance test (OGTT) in n-STZ diabetic rats. [A] Line curves depict the changes in blood glucose response in normal control, diabetic control, and diabetic treated groups [Galan 5 mg/kg; Vilda 30 mg/kg; or their combination, after 0, 30, 60, 90 and 120 min following administration of glucose (2.5 g/kg, p.o)]. All treatments were gavaged orally for four weeks. Values are means of 10 rats ± S.E.M. [B] Area under the curve (AUC) of the OGTT to compare between different studied groups. As compared with normal control (*), diabetic control (#), Galan5 (η), and Vilda30 (Ψ)-treated groups (one-way ANOVA followed by Tukey post hoc test) at *P*< 0.05.

The fasting blood glucose level of the diabetic rats increased by 2.3 folds compared to that in the normal animals. In the latter, the glucose level reached its peak half an hour after the oral administration of glucose then decreased gradually to the fasting level thereafter. However, in the diabetic animals, the blood glucose was elevated by about 190% above the normal one after 30 min. and failed to reach the pre-prandial level 2 hours later, confirming, thus, a state of impaired glucose tolerance (IGT) (Table 2 and Fig 1A). The different treatment regimens abated the high glucose level as confirmed by the AUC (Fig 1B).

**Table 2:** Effect of different treatments on oral glucose tolerance test (OGTT) in n-STZ diabetic rats.

Experimental Groups	Blood glucose level (mg/dl)				
	0 minutes	30 minutes	60 minutes	90 minutes	120 minutes
Normal control	87.9 ± 3.12	120.7 ± 3.59	114.8 ± 4.22	102.5 ± 3.06	85.7 ± 3.43
Diabetic control	202.3 ± 7.20*	350.4 ± 9.00*	308.7 ± 6.3*	276.0 ± 10.00*	230.0 ± 9.84*
Galan 2.5	143.4 ± 4.05* ^**#**^	237.8 ± 5.09* ^**#**^	227.0 ± 12.60* ^**#**^	172.8 ± 9.61* ^**#**^	153.1 ± 5.18* ^**#**^
Galan 5	128.2 ± 4.67* ^**#**^	250.6 ± 11.57* ^**#**^	229.6 ± 12.55* ^**#**^	179.9 ±13.69* ^**#**^	134.5 ± 2.93* ^**#**^
Galan 10	125.3 ± 5.37* ^**#**^	240.6 ± 8.88* ^**#**^	213.3 ± 11.97* ^**#**^	171.0 ± 7.32* ^**#**^	131.2 ± 5.53* ^**#**^
Vilda 3	178.9 ± 4.99* ^#Ψ^	349.8 ± 16.62* ^Ψ^	295.6 ± 13.74* ^Ψ^	236.8 ± 9.56* ^**#**^	198.7 ± 7.35* ^#Ψ^
Vilda 10	139.9 ± 6.24*^#^	292.8 ± 12.17* ^**#**^	251.0 ± 9.41* ^**#**^	218.2 ± 6.50* ^**#**^	162.6 ± 3.63* ^#Ψ^
Vilda 30	122.5 ± 4.9*^#^	273.6 ± 7.32* ^**#**^	247.3 ± 6.95* ^**#**^	205.6 ± 8.55* ^**#**^	134.2 ± 3.72* ^**#**^
Galan 5+Vilda 30	109.9 ± 4.8^#ηΨ^	166.6 ± 6.44* ^#ηΨ^	153.5 ± 5.52* ^#ηΨ^	132.0 ± 5.5* ^#ηΨ^	119.3 ± 4.73* ^#ηΨ^

Galantamine (Galan 2.5, 5 & 10 mg/kg), vildagliptin (Vilda 3, 10 & 30 mg/kg) and their combination (Galan 5 & Vilda 30) were gavaged orally for four weeks. Values are means of 10 rats ± S.E.M as compared with normal control (*), diabetic control (#), Galan5 (η), and Vilda30 (Ψ)-treated groups (one-way ANOVA followed by Tukey post hoc test) at *P*< 0.05.

### Glucose Homeostasis-Related Parameters

**Fig 2:**
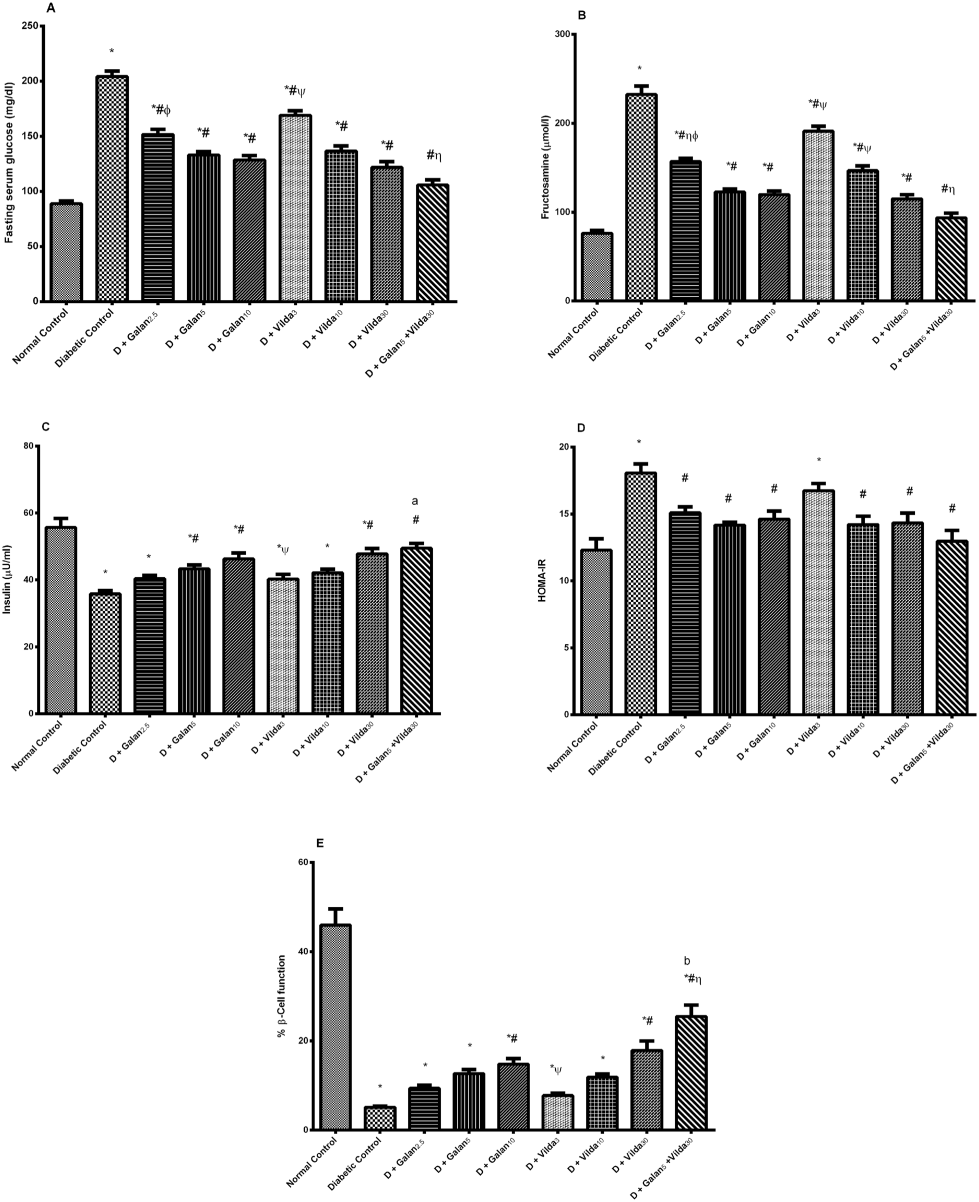
Effect of galantamine and/or vildagliptin on glucose homeostasis. Effect of different doses of galantamine (Galan 2.5, 5 & 10 mg/kg), vildagliptin (Vilda 3, 10 & 30 mg/kg) and their combination (Galan 5 + Vilda 30) on the fasting serum glucose (A), fructosamine (B), insulin (C), HOMA-IR (D), % β-cell function (E) in n5-STZ diabetic rats. Drugs were gavaged orally for four weeks. Values are means of 10 rats ± S.E.M as compared with normal control (*), diabetic control (#), Galan5 (η), Galan10 (ɸ) and Vilda30 (Ψ)-treated groups (one-way ANOVA followed by Tukey post hoc test) at *P*< 0.05. (a) Additive and (b) synergistic interaction when Galan5 and Vilda30 were combined using Factorial Design.

As shown in Fig 2, treatment with galantamine and vildagliptin reduced the fasting serum glucose (A), fructosamine (B) and HOMA-IR (D) *vs*. the diabetic group (*P* < 0.001). Additionally, in a dose dependent manner, both treatments have increased the fasting serum insulin (C) and % β-cell function (E). The drug combination regimen showed the best improving effect on almost all the aforementioned parameters reaching to an additive or synergistic level in some cases. It should be noted that in case of serum glucose level, the combined treatment interaction did not reach an additive level, i.e. did not cause hypoglycemia, pointing to the safe use of this combination.

### Lipid Profile-Related Parameters

**Fig 3:**
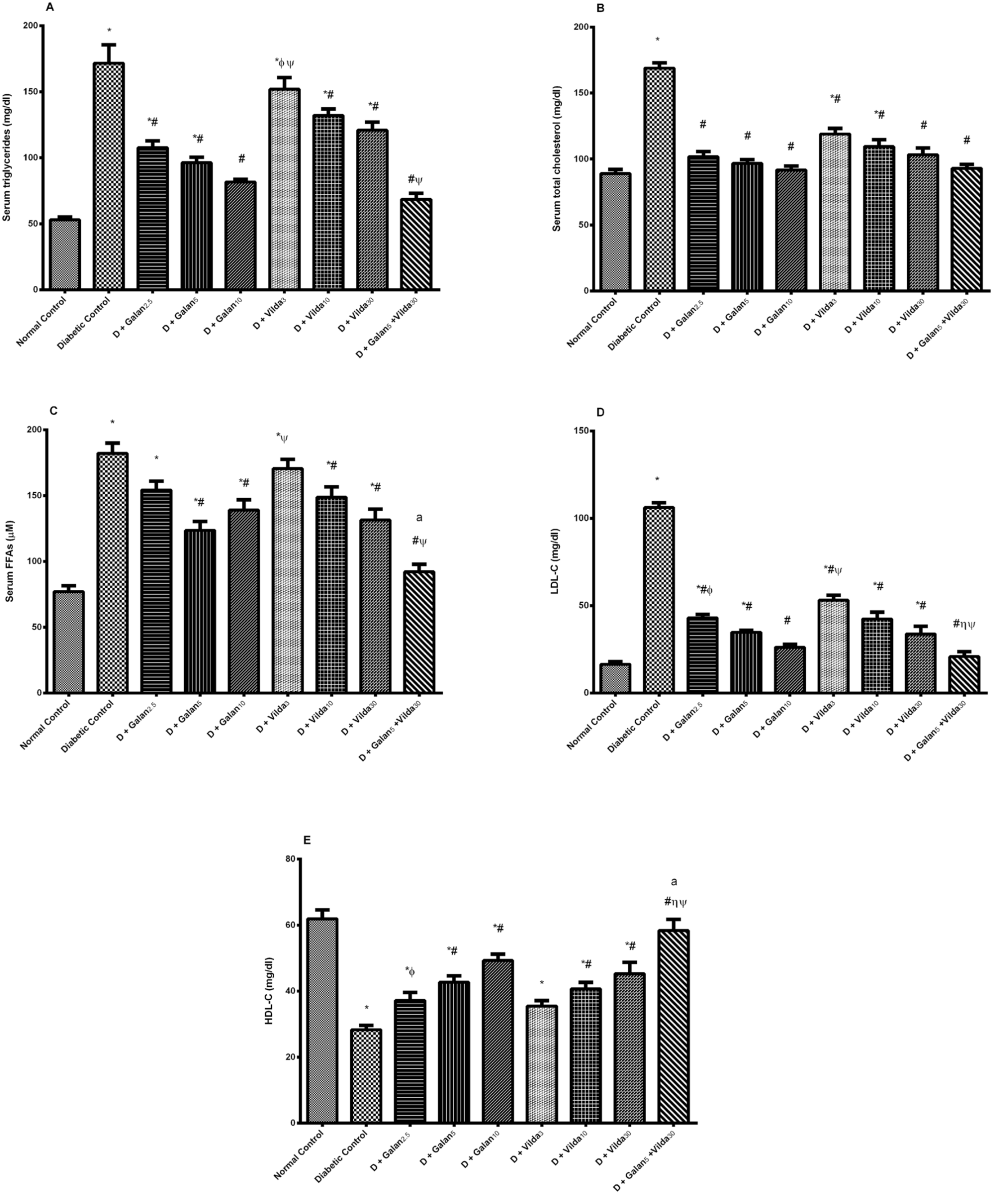
Effect of galantamine and/or vildagliptin on serum lipid profile. Effect of different doses of galantamine (Galan 2.5, 5 & 10 mg/kg), vildagliptin (Vilda 3, 10 & 30 mg/kg) and their combination (Galan 5 + Vilda 30) on the serum triglycerides (A), total cholesterol (B), free fatty acids (FFAs) (C), LDL-C (D), and HDL-C (E) in n5-STZ diabetic rats. Drugs were gavaged orally for four weeks. Values are means of 10 rats ± S.E.M as compared with normal control (*), diabetic control (#), Galan5 (η), Galan10 (ɸ) and Vilda30 (Ψ)-treated groups (one-way ANOVA followed by Tukey post hoc test) at *P*< 0.05. (a) Additive interaction when Galan5 and Vilda30 were combined using Factorial Design.

**Fig 4:**
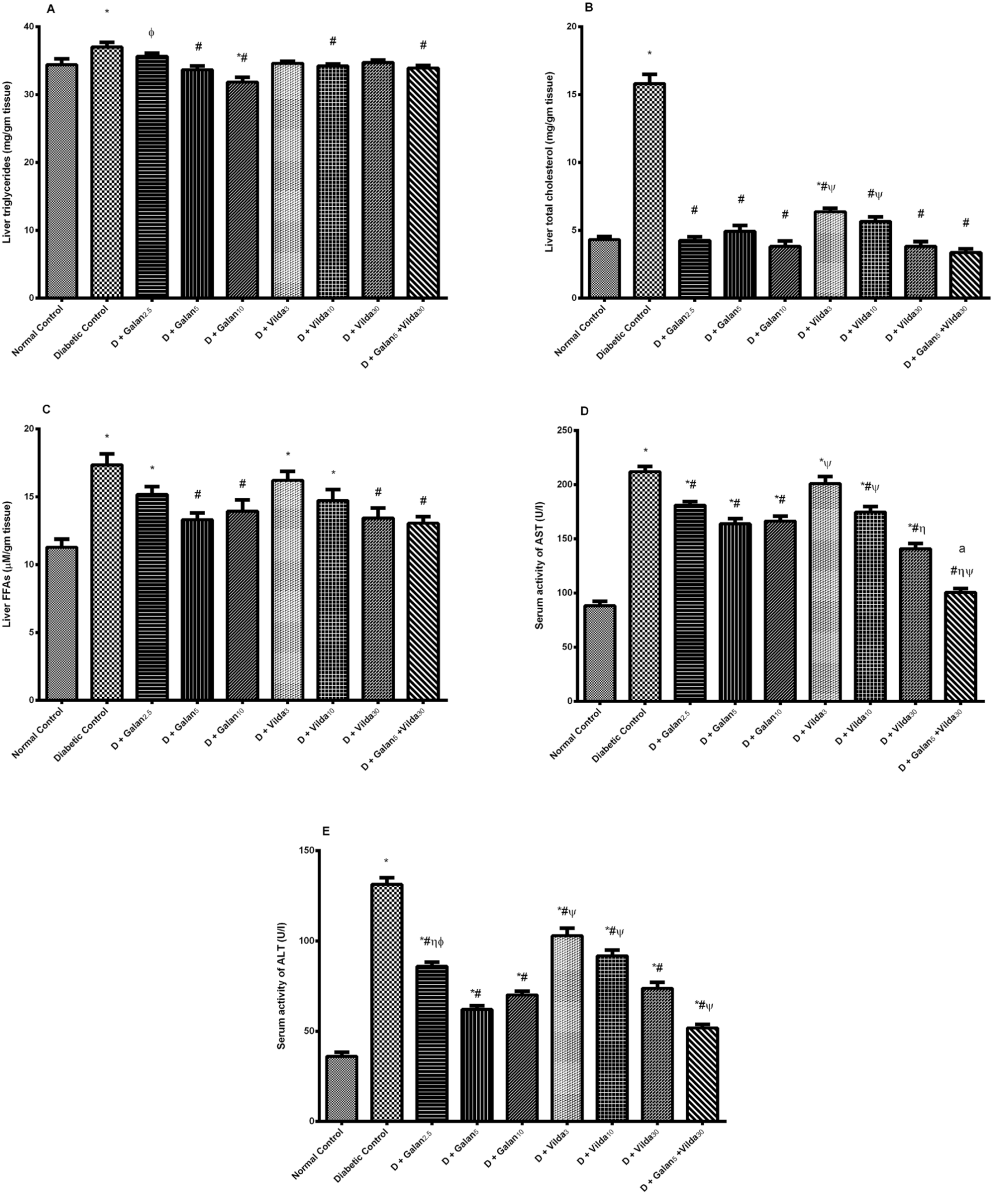
Effect of galantamine and/or vildagliptin on hepatic lipid profile. Effect of different doses of galantamine (Galan 2.5, 5 & 10 mg/kg), vildagliptin (Vilda 3, 10 & 30 mg/kg) and their combination (Galan 5 + Vilda 30) on the hepatic triglycerides (A), total cholesterol (B), free fatty acids (FFAs) (C), as well as serum activities of AST (D), and ALT (E) in n-STZ diabetic rats. Drugs were gavaged orally for four weeks. Values are means of 10 rats ± S.E.M as compared with normal control (*), diabetic control (#), Galan5 (η), Galan10 (ɸ) and Vilda 30 (Ψ)-treated groups (one-way ANOVA followed by Tukey post hoc test) at *P*< 0.05. (a) Additive interaction when Galan5 and Vilda30 were combined using Factorial Design.

**Fig 5:**
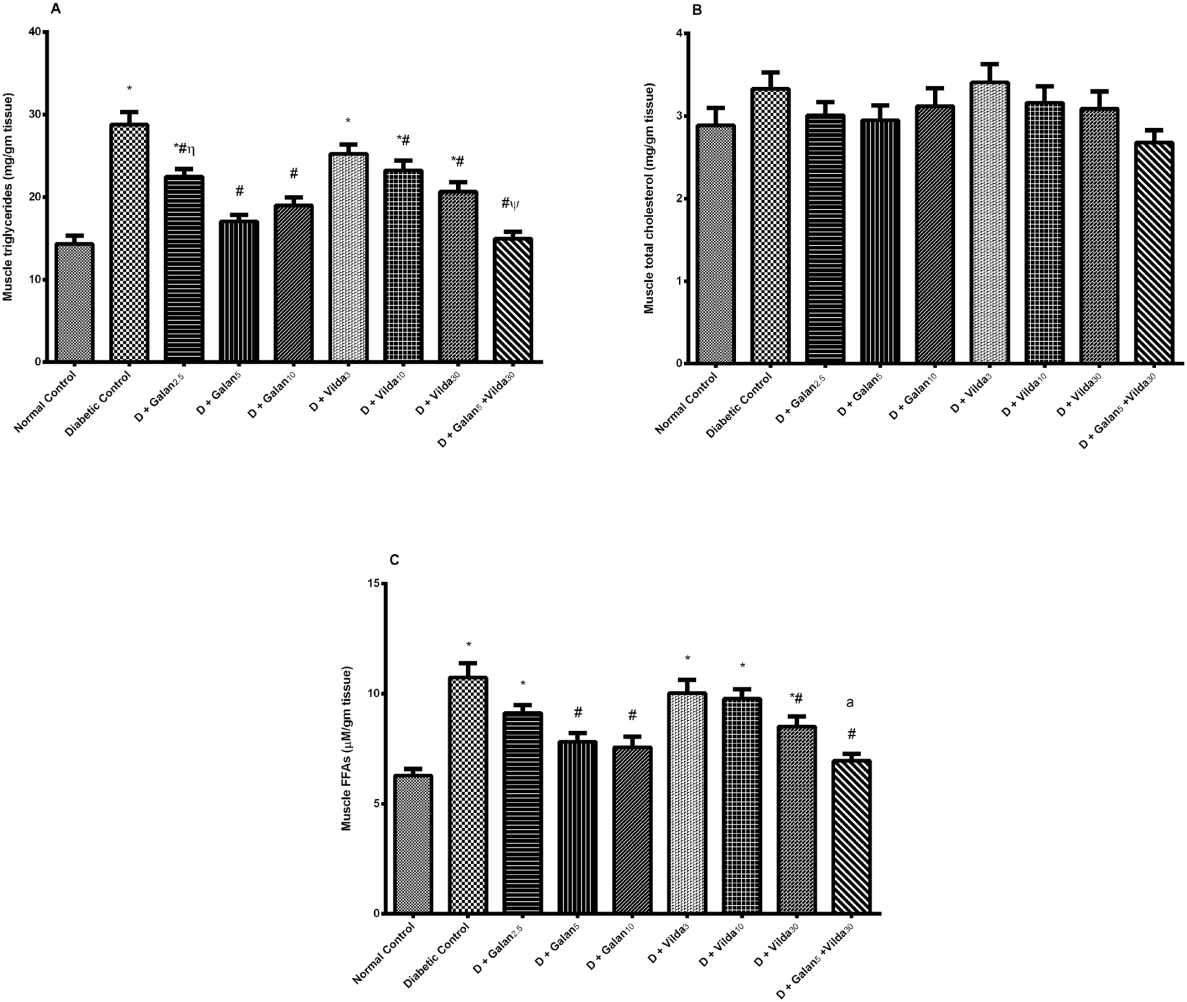
Effect of galantamine and/or vildagliptin on muscular lipid profile. Effect of different doses of galantamine (Galan 2.5, 5 & 10 mg/kg), vildagliptin (Vilda 3, 10 & 30 mg/kg) and their combination (Galan 5 + Vilda 30) on the muscular triglycerides (A), total cholesterol (B), and free fatty acids (FFAs) (C), in n-STZ diabetic rats. Drugs were gavaged orally for four weeks. Values are means of 10 rats ± S.E.M as compared with normal control (*), diabetic control (#), Galan5 (η), and Vilda30 (Ψ)-treated groups (one-way ANOVA followed by Tukey post hoc test) at *P*< 0.05. (a) Additive interaction when Galan5 and Vilda30 were combined using Factorial Design.

The n5-STZ model elevated markedly serum TGs, TC, FFAs, and LDL-C, but decreased that of HDL-C Fig 3A–3E. Nevertheless, both treatments improved significantly the altered lipid panels in a dose dependent manner, with the combined regimen showing the best effect, reaching the normal level in most of the previously mentioned parameters. In the liver (Fig 4), the diabetic model caused a subtle, yet significant elevation in TGs (A), with marked increment in the levels of TC (B), FFAs (C), as well as serum AST (D) and ALT (E); however, all treatment interventions lowered these biomarkers. Muscular contents of TGs and FFAs showed almost the same pattern; they were elevated significantly in the diabetic group (Fig 5A and 5C), while treatment with galantamine (5 and 10 mg/ kg) and the combination regimen normalized them. Nevertheless, TC content (B) was not altered in any of the tested groups.

### Changes in AChE Activity in Brain, Liver, and Muscle

**Fig 6:**
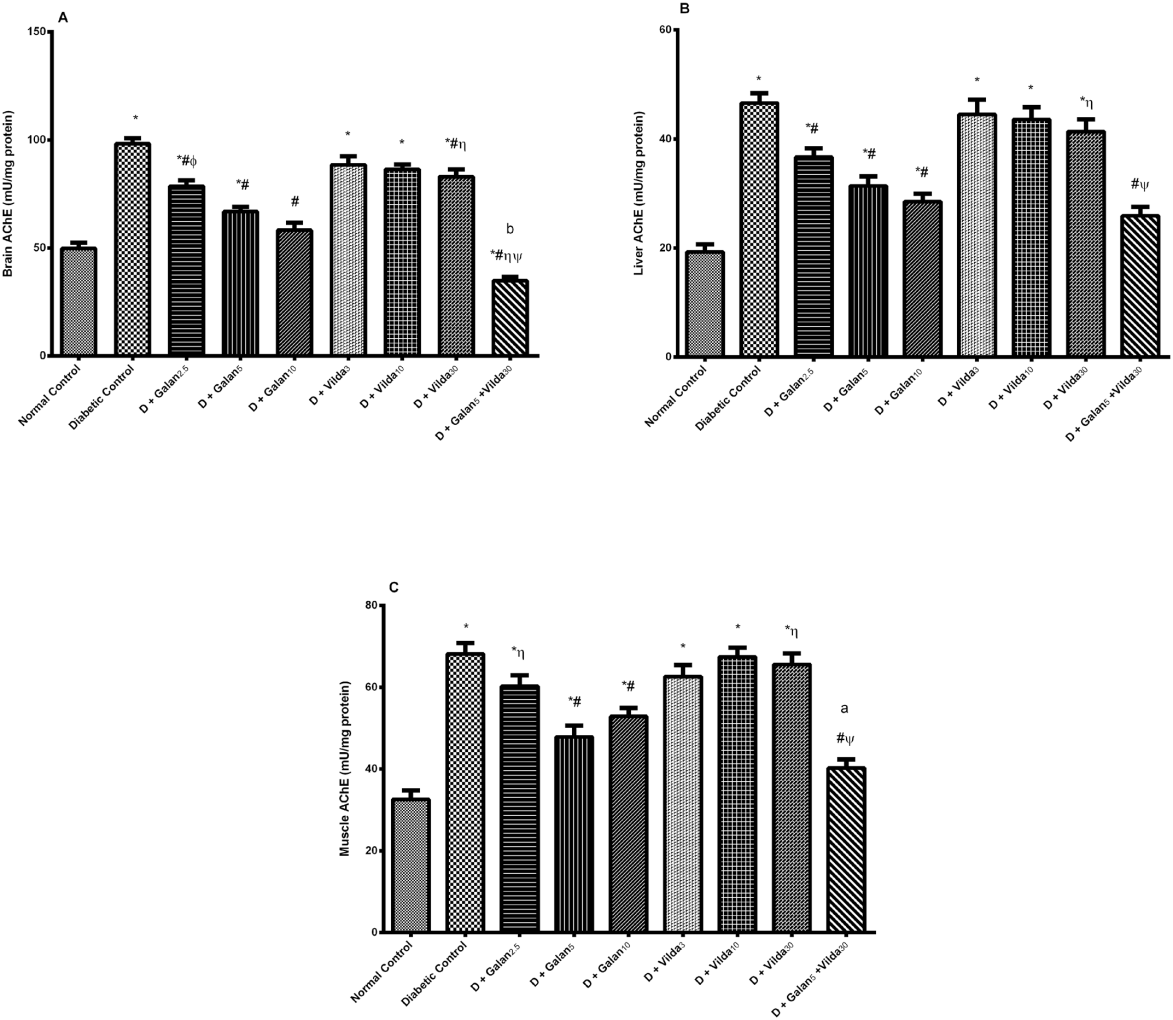
Effect of galantamine and/or vildagliptin on AChE activity. Effect of different doses of galantamine (Galan 2.5, 5 & 10 mg/kg), vildagliptin (Vilda 3, 10 & 30 mg/kg) and their combination (Galan 5 + Vilda 30) on the acetylcholinesterase (AChE) activity in brain (A), liver (B) and muscle (C) in n-STZ diabetic rats. Drugs were gavaged orally for four weeks. Values are means of 10 rats ± S.E.M as compared with normal control (*), diabetic control (#), Galan5 (η), Galan10 (ɸ) and Vilda30 (Ψ)-treated groups (one-way ANOVA followed by Tukey post hoc test) at *P*< 0.05. (b) Synergistic and (c) potentiating interaction when Galan 5 and Vilda 30 were combined using Factorial Design.

The AChE activity (Fig 6) was markedly elevated in the brain (A), liver (B) and muscle (C) of the diabetic group to reach 1.9, 2.4 and 2.1 folds, respectively, in comparison with the normal control values. Galantamine dose dependently abated AChE activity in the tested organs, while the vildagliptin effect was limited to the brain enzyme. The lowering effect mediated by the combination regimen, was the utmost in the three organs, where vildagliptin potentiated the galantamine effect in the periphery and interacted synergistically in the brain.

### Changes in Oxidative Stress Parameters (Nrf2, TAC, and MDA) in Liver and Muscle

**Fig 7:**
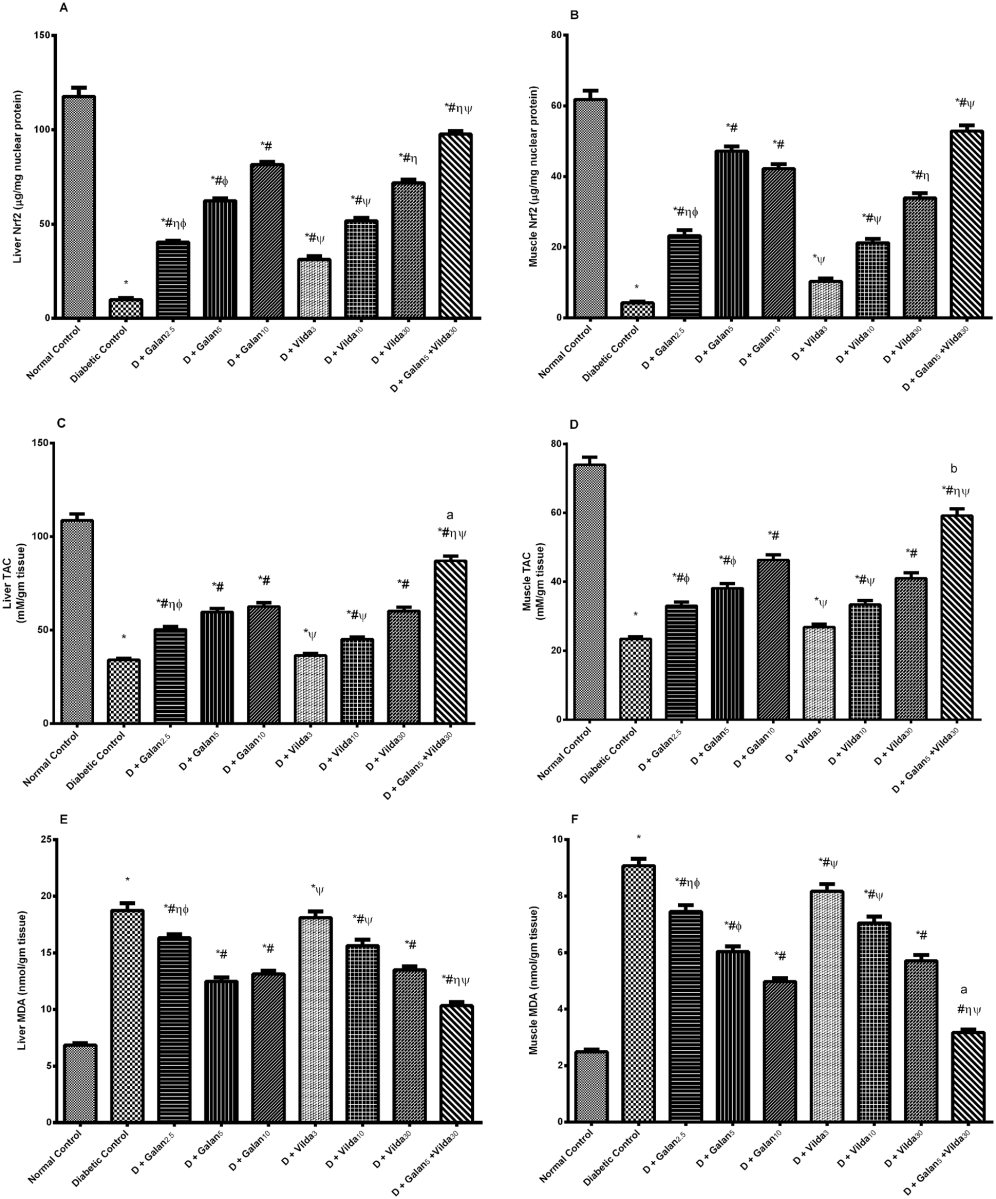
Effect of galantamine and/or vildagliptin on oxidative stress parameters. Effect of different doses of galantamine (Galan 2.5, 5 & 10 mg/kg), vildagliptin (Vilda 3, 10 & 30 mg/kg) and their combination (Galan 5 + Vilda 30) on hepatic (A) and muscular (B) transcription activation of Nrf2 (nuclear factor-erythroid-2-related factor 2), total antioxidant capacity (C, D) and malondialdehyde (MDA) content (E, F), respectively, in n-STZ diabetic rats. Drugs were gavaged orally for four weeks. Values are means of 10 rats ± S.E.M as compared with normal control (*), diabetic control (#), Galan5 (η), Galan10 (ɸ) and Vilda30 (Ψ)-treated groups (one-way ANOVA followed by Tukey post hoc test) at *P*< 0.05. (a) Additive and (b) synergistic interactions when Galan5 and Vilda30 were combined using Factorial Design.

The n5-STZ model caused a tremendous decrease in the Nrf2 transcription activation (Fig 7A and 7B) and TAC (C, D) in the two organs tested, as compared to normal control rats (*P* < 0.001). These changes were reversed in the treated groups in a dose dependent manner and with the greatest increase observed in the combination group (*P* < 0.001). Contrariwise, the diabetic model increased the MDA content in the liver and muscle homogenates (E, F), as compared to the normal rats, whereas the different doses of galantamine and vildagliptin decreased it significantly. Once again, the greatest inhibitory effect was observed in the combination treated group.

### Changes in Hepatic and Muscular Apoptosis Biomarkers (Caspase-3 and Cytochrome c)

**Fig 8:**
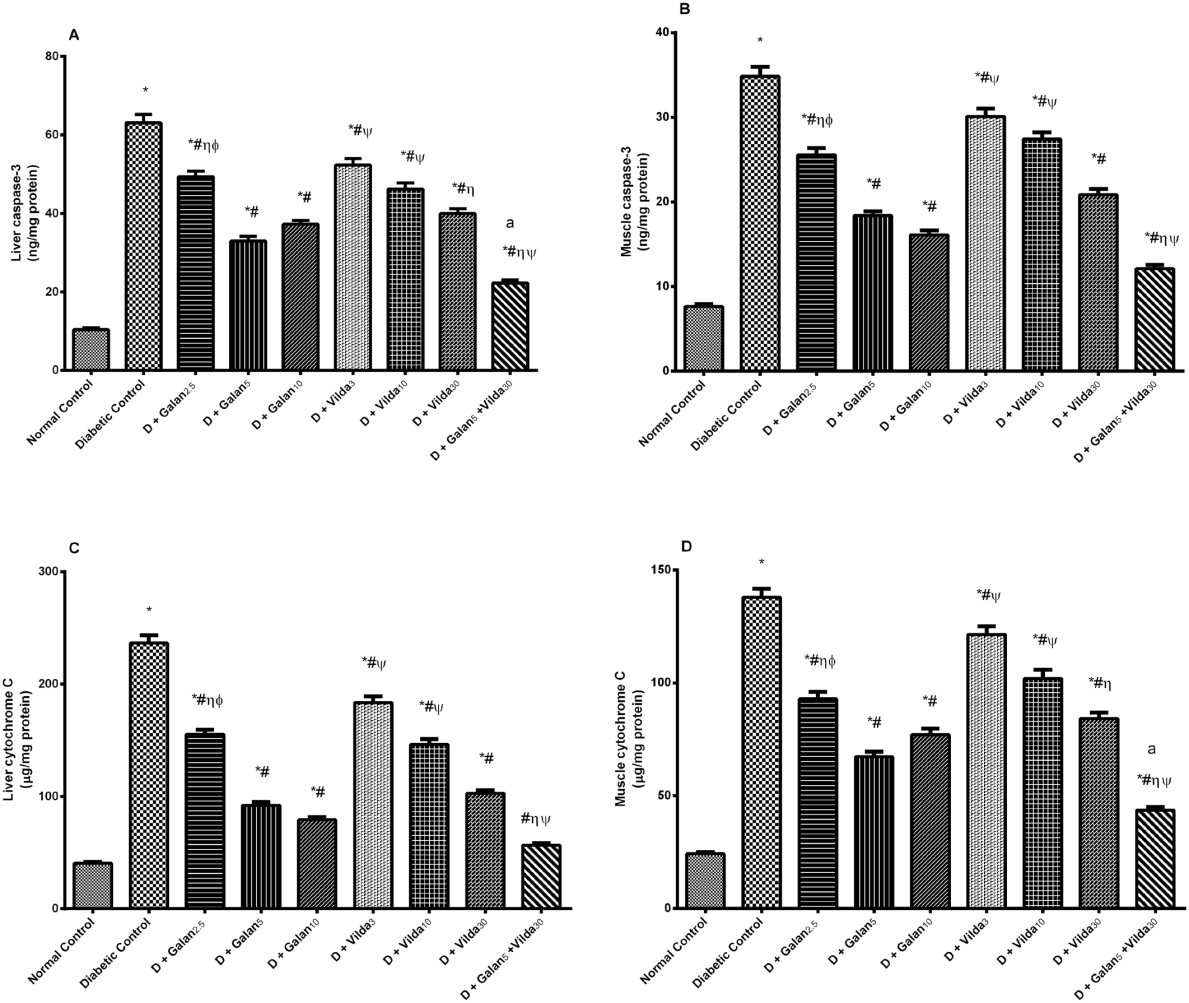
Effect of galantamine and/or vildagliptin on apoptosis biomarkers. Effect of different doses of galantamine (Galan 2.5, 5 & 10 mg/kg), vildagliptin (Vilda 3, 10 & 30 mg/kg) and their combination (Galan 5 + Vilda 30) on hepatic and muscular levels of caspase-3 (A, B) and cytochrome c (C, D), respectively, in n-STZ diabetic rats. Drugs were gavaged orally for four weeks. Values are means of 10 rats ± S.E.M as compared with normal control (*), diabetic control (#), Galan5 (η), Galan10 (ɸ) and Vilda30 (Ψ)-treated groups (one-way ANOVA followed by Tukey post hoc test) at *P* < 0.05. (a) Additive interaction when Galan5 and Vilda30 were combined using Factorial Design.

Fig 8 showed that hepatic and muscular levels of caspase-3 (A, B) and cytochrome c (C, D) were boosted in the diabetic rats relative to the normal control (*P* < 0.001), while the different regimens lowered them in a dose dependent manner. The lowering effect of galantamine was better than that of vildagliptin and was the lowest in the combination treated group.

### Inflammatory Mediators and Adipokines

**Fig 9:**
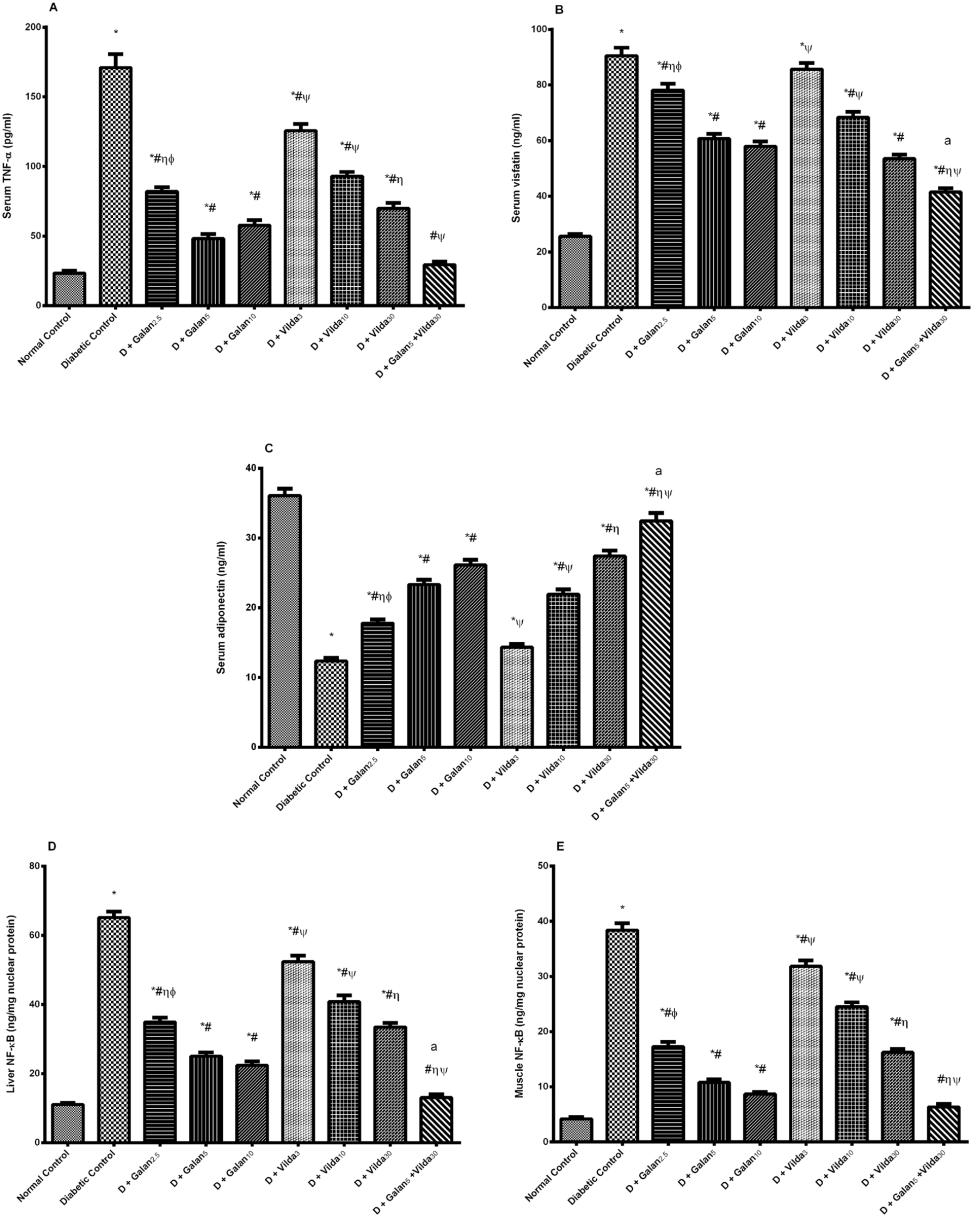
Effect of galantamine and/or vildagliptin on inflammatory mediators and adipokines. Effect of different doses of galantamine (Galan 2.5, 5 & 10 mg/kg), vildagliptin (Vilda 3, 10 & 30 mg/kg) and their combination (Galan5 + Vilda 30) on serum TNF-α (A), visfatin (B) and adiponectin (C), as well as hepatic (D) and muscular (E) nuclear factor κB (NF-κB) in n-STZ diabetic rats. Drugs were gavaged orally for four weeks. Values are means of 10 rats ± S.E.M as compared with normal control (*), diabetic control (#), Galan5 (η), Galan10 (ɸ) and Vilda30 (Ψ)-treated groups (one-way ANOVA followed by Tukey post hoc test) at *P*< 0.05. (a) Additive interaction when Galan5 and Vilda30 were combined using Factorial Design.

As shown in Fig 9, n5-STZ model increased significantly serum TNF-α (7.3 folds; A) and visfatin (3.5 folds; B), as well as hepatic (D) and muscular (E) NF-κB, but decreased that of serum adiponectin (C), relative to the normal ones (*P*< 0.001). Nevertheless, both treatments amended the inflammatory mediators in a dose dependent manner; anew the combination regimen showed the best effect.

### Insulin Signaling Pathway

**Fig 10:**
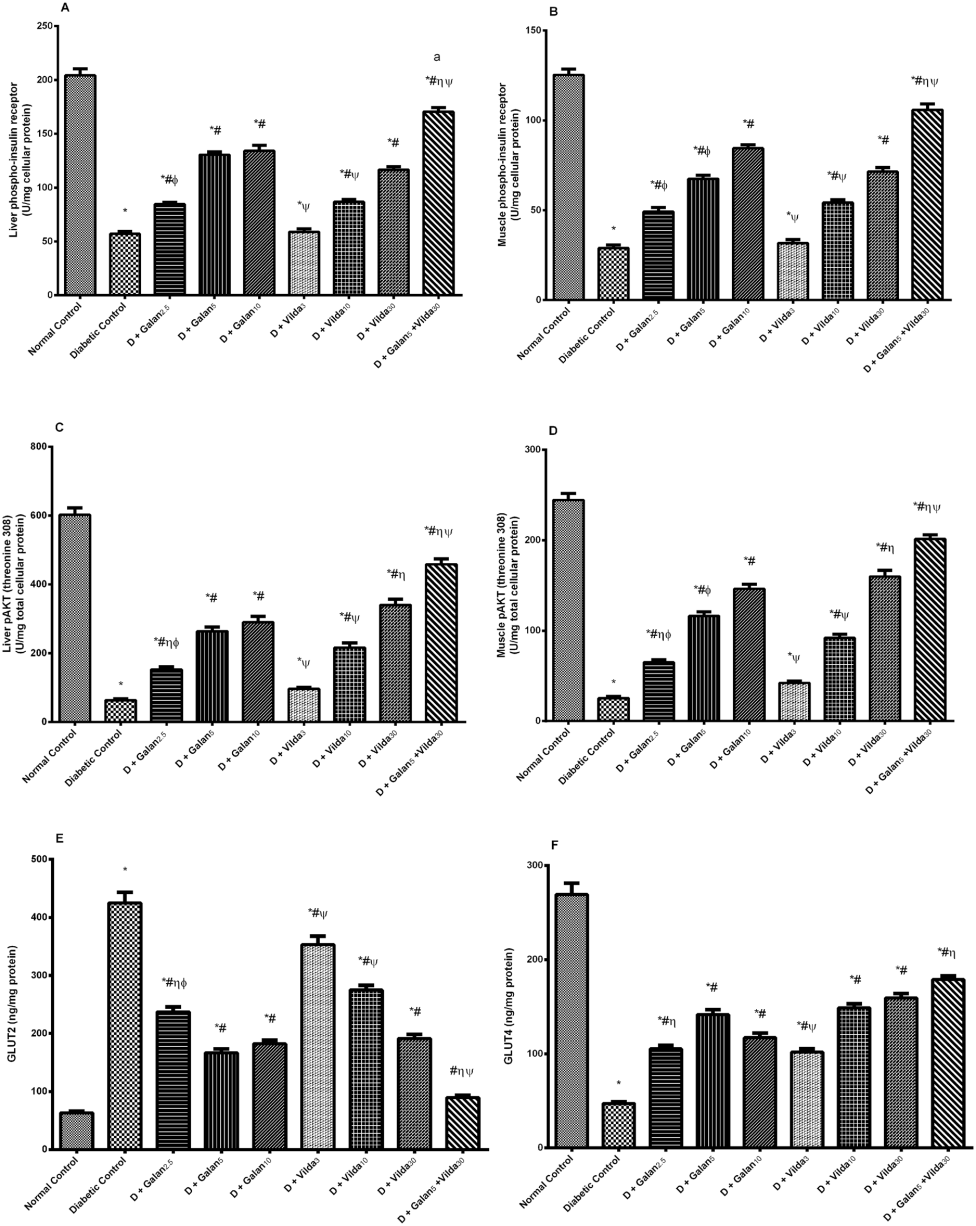
Effect of galantamine and/or vildagliptin on insulin signaling pathway. Effect of different doses of galantamine (Galan 2.5, 5 & 10 mg/kg), vildagliptin (Vilda 3, 10 & 30 mg/kg) and their combination (Galan5 + Vilda 30) on hepatic and muscular contents of phosphorylated insulin receptor (A, B), and p-Akt (C, D), respectively, as well as hepatic GLUT2 (E) and muscular GLUT4 (F) in n-STZ diabetic rats. Drugs were gavaged orally for four weeks. Values are means of 10 rats ± S.E.M as compared with normal control (*), diabetic control (#), Galan5 (η), Galan10 (ɸ) and Vilda30 (Ψ)-treated groups (one-way ANOVA followed by Tukey post hoc test) at *P*< 0.05. (a) Additive interaction when Galan5 and Vilda30 were combined using Factorial Design.

**Fig 11:**
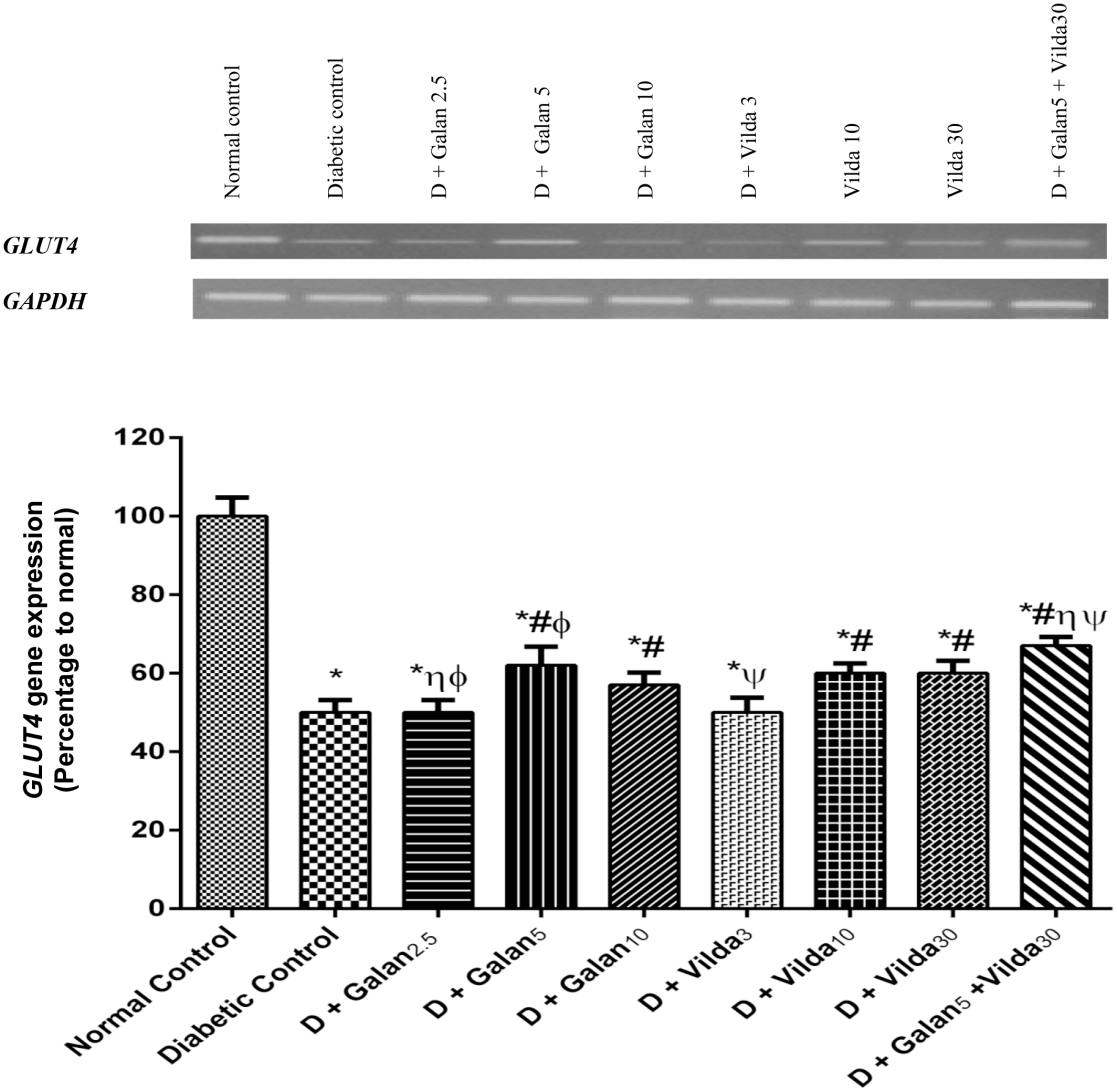
Effect of galantamine and/or vildagliptin on *GLUT4* gene expression. Effect of different doses of galantamine (Galan 2.5, 5 & 10 mg/kg), vildagliptin (Vilda 3, 10 & 30 mg/kg) and their combination (Galan5 + Vilda 30) on muscular *GLUT4* gene expression in n-STZ diabetic rats. The upper panel represents stained agarose gels of RT-PCR products as a representative of one rat for each group. Data in the lower panel represents means of 10 rats ± S.EM as compared with normal control (*), diabetic control (#), Galan5 (η), Galan10 (ɸ) and Vilda30 (Ψ)-treated groups (one-way ANOVA followed by Tukey post hoc test) at *P*< 0.05.

As presented in Fig 10, the n5-STZ model caused a tremendous decrease in the hepatic/muscular phosphorylated insulin receptor (A, B), p-Akt (C, D), hepatic GLUT2 (E), and muscular GLUT4 (F) compared to normal control rats. These effects were reversed almost in all treated groups dose dependently, with the combination regimen showing the best effect (*P*< 0.001). Regarding the expression of *GLUT4* gene (Fig 11), diabetes halved its expression, while different treatment regimens induced it significantly (*P*< 0.05).

### Wnt/β-Catenin Pathway

**Fig 12:**
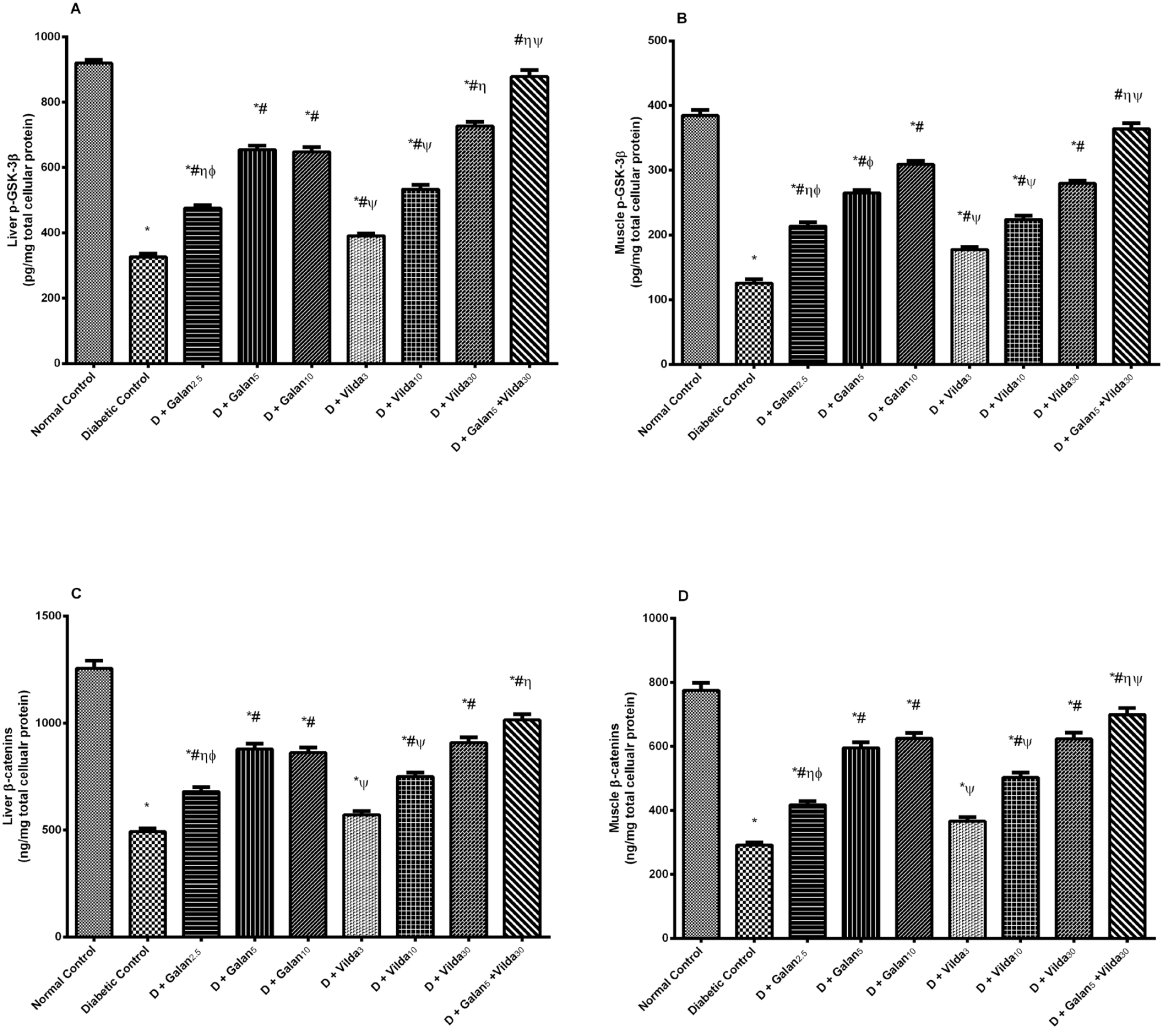
Effect of galantamine and/or vildagliptin on Wnt/β-catenin pathway. Effect of different doses of galantamine (Galan 2.5, 5 & 10 mg/kg), vildagliptin (Vilda 3, 10 & 30 mg/kg) and their combination (Galan5 + Vilda 30) on the hepatic and muscular contents of p-GSK-3β (A, B) and β-catenin (C, D) in n-STZ diabetic rats. Drugs were gavaged orally for four weeks. Values are means of 10 rats ± S.E.M as compared with normal control (*), diabetic control (#), Galan5 (η), Galan10 (ɸ) and Vilda30 (Ψ)-treated groups (one-way ANOVA followed by Tukey post hoc test) at *P*< 0.05. (a) Additive and (b) synergistic interactions when Galan5 and Vilda30 were combined using Factorial Design.

The hepatic and muscular contents of p-GSK-3β and total β-catenin were significantly decreased in the untreated diabetic group, compared to the control one, respectively (Fig 12A–12D). These major effectors of the Wnt pathway were corrected to different extents by the treatment regimens (*P*< 0.05), with the combination regimen showing the best effect among the other treated groups (*P*< 0.001).

I/R, presented as HOMA-IR, correlated positively with AChE and NF-κB, but negatively with Nrf2, transcription activation, p-GSK-3β and β-catenin (Table 3).

**Table 3:** Correlation between HOMA-IR with acetylcholinesterase (AChE) activity, Nrf2 transcription activity, NF-κB, p-GSK-3β, and β-catenin.

	HOMA-IR	
	r	*P*
Brain AChE activity	0.828*	0.003
Liver AChE activity	0.801*	0.005
Muscle AChE activity	0.068*	0.031
Liver Nrf2 transcription activity	-0.686*	0.029
Muscle Nrf2 transcription activity	-0.735*	0.015
Liver NF-κB	0.817*	0.004
Muscle NF-κB	0.859*	0.001
Liver GSK-3β	-0.728*	0.017
Muscle GSK-3β	-0.655*	0.04
Liver β-catenin	-0.686*	0.029
Muscle β-catenin	-0.780*	0.008

Correlation was carried out in the untreated and treated n-STZ diabetic rats, (*) indicates significant difference at *P*< 0.05

## Discussion

Diabetes mellitus is documented to correlate with cognitive dysfunctions and CNS abnormalities. Although the exact mechanism is not fully understood, the variation in AChE activity, an enzyme that has a fundamental role in learning and memory, can play a profound role [3, 29]. In addition, AChE was found in tissues devoid of cholinergic innervations, indicating its potential non-cholinergic function, including response to stress and neurogenesis [30].

In the present work, n5-STZ model activated the AChE enzyme in brain, liver and muscle tissues of the diabetic rats, while the administration of the cholinesterase inhibitor galantamine reduced it in all tissues, as expected; this effect points to a possible antidiabetic mechanism of galantamine through the inhibition of AChE enzyme. Nevertheless, vildagliptin showed a subtle, yet significant reduction in the cerebral enzyme only. Although vildagliptin cannot pass the BBB, yet GLP-1 can reach the brain to act as the effector molecule that decreases the AChE activity, possibly *via* activating its central receptor. Addition of vildagliptin to galantamine showed a marked decrease in AChE activity that was less than the highest dose of galantamine in all tissues tested and even less than the normal level in the brain. These effects can highlight the possible co-stimulation of the vagal tone by combining the two drugs *via* inhibiting AChE.

The n5-STZ model develops the classical diabetic picture of T2DM [31], where it elevated fructosamine, sharply reduced pancreatic insulin stores with the consequent decrease in basal insulin, besides polyphagia and polydipsia that are aggravated by I/R in adulthood. The polyphagia/accentuated weight gain, witnessed in the diabetic rats, can be attributed to the STZ-induced low insulin level and/or to the CNS insulinopenia mediated by the peripheral I/R. This metabolic change minimizes insulin uptake at the BBB [32], which is altered by hyperglycemia [33], leading to uncontrolled food intake. One of the fundamental actions of the brain insulin, derived mainly from the pancreatic *β*-cells, is to regulate the food consumption [1]. As a support, Chistyakova *et al*. [34] owed the cognitive deficit in rats with neonatal T2DM to the reported low brain insulin concentration and impaired insulin receptor substrate-2-mediated signaling pathways in the brain.

On the other hand, galantamine dose dependently decreased food intake in diabetic rats to mimic the report of Satapathy *et al*. [5] in obese mice. This effect can be accredited, on one hand, to the improved insulin level, observed herein, and on the other hand, to the stimulation of the vagal tone, which is documented to decrease appetite, food consumption and weight gain [35]. Moreover, stimulation of the presynaptic alpha7 nicotinic acetylcholine receptor (α7nAChR) by galantamine emphasizes the importance of the central cholinergic signaling in controlling food intake [5, 36, 37]. Conversely, vildagliptin had no influence on food intake nor weight gain, results that coincide with those of Amori *et al*. [38], who stated that DPP-4 inhibitors are weight neutral.

Apart from its central effects, galantamine, in the current work, corrected the glucose homeostasis-related parameters. In case of obesity [39] and diabetes mellitus [40], the vagal tone is suppressed and the pathway is dysfunctional. Therefore, the antidiabetic effects of galantamine can be referred to stimulation of the cholinergic pathway, being an inhibitor of the AChE, an agonist of the α7nAChR, besides activating the efferent vagus nerve [5]. The latter serves as the neuronal pathway in the cross-talk between liver, pancreatic β-cells and adipose tissue, to modulate insulin secretion, pancreatic β-cell mass, energy expenditure regulation, glucose metabolism, hepatic glucose/glycogen production, systemic insulin sensitivity and fat distribution between liver and peripheral tissues [5, 41].

Vildagliptin mediates its antidiabetic effect by inhibiting DPP-4 to augment intact GLP-1 [42], improve β-cell function and mass [43], increase insulin secretion, and reduce glucose excursions [44]. These facts are further confirmed by our findings and may be assigned to the activation of GLP-1 receptor expressed on the terminals of vagal afferent fibers innervating the GIT to promote insulin secretion and to improve glucose profile, putatively *via* a vago-vagal reflex [45]. The best-corrected glucose profile was seen in the combination-treated group; whether this effect is ascribed, even partly, to the activation of the vagus tone remains to be elucidated.

The current study tested, for the first time, the influence of the two drugs on the insulin signaling trail. Apart from the classical mechanism of action, both galantamine and vildagliptin increased the phosphorylation of insulin receptors with the consequent activation/phosphorylation of Akt and the elevation of GLUT2 and GLUT4, effects that are responsible for the improved insulin sensitivity. Phosphorylated insulin receptors and Akt corrects the function of GLUT2 in the liver to enhance glucose uptake on one side and to block gluconeogenesis and mediate glycogen synthesis on the other side [46]. This pathway also enhances the translocation of GLUT4 to the cell membrane surface of skeletal muscle to increase muscular glucose uptake.

Another pathway that was tackled here is the Wnt/β-catenin; again, both drugs enhanced this pathway when tested in liver and muscle, indicating another pathway for improvement of insulin sensitivity. An earlier study [8] stated that the development of type 2 diabetes results from abnormalities in the Wnt signaling pathway, which regulates hormone gene expression and metabolic homeostasis [47, 48], supporting, thus, the present findings in the n5-STZ animals. On the contrary, the two drugs increased the phosphorylated/inactivate GSK-3β and hence, prevented the proteosomal degradation of β-catenin. The salvaged β-catenin is translocated to the nucleus to team up with TCF and activates Wnt target gene expression. Previously, Jin et al. [48] pointed to the positive influence of insulin, whose level was corrected by both drugs herein, on the dimerization of β-catenin with TCF, which is important for glucose and lipid metabolism. Moreover, in a cross link between the two studied pathways, Abiola et al. [49] found that Wnt signaling also stimulated GLUT4 translocation to the plasma membrane independent of insulin, and also restored insulin sensitivity in insulin-resistant myotubes.

Wnt pathway also plays a role in mediating incretin hormones functions [50], which can add to the vildagliptin antidiabetic mechanism. Such pathway regulates the synthesis of proglucagon and consequently of GLP-1 and GIP *via* the bipartite transcription factor β-catenin/TCF [51].

Pan dyslipidemia, in serum, liver and skeletal muscle, was another I/R feature that was successfully negated by galantamine, findings that were studied for the first time in a diabetic model. These effects can be owed to the parasympathomimetic/anticholinesterase effects of galantamine, where the autonomic nervous system plays a role in the fine-tuning of the hepatic lipid metabolism regulation [52]. Obese children were reported to have low parasympathetic tone associated with hypertriglyceridemia, hyperglycemia and I/R [53], and its activation leads to decreased lipolysis [54]. These facts support the current findings, where serum TGs were elevated in the current model and lowered by galantamine, an effect that was more apparent on serum TGs than the hepatic ones.

Moreover, the improvement of the deranged insulin signaling, with the consequent low glucose level by galantamine, lends another explanation for the corrected lipid panel observed herein. Previously, hyperglycemia was reported to increase de novo lipogenesis markedly by increasing sterol regulatory element-binding protein-1c (SREBP-1c) [49]. Although the effect of galantamine on SREBP-1c was not tested here, yet an early study [55] reported a unique relationship between lipid homeostasis, the lipid-sensitive transcription factor, SREBP-1c, and the parasympathetic response in cardiomyocytes. The authors found that the cardiomyocytes of SREBP-1c KO mice responded far less to the parasympathomimetic drug, carbamylcholine, compared with the wild type animals. The activation of the Wnt signaling by galantamine can additionally verify this assumption, where inhibited GSK-3β and activated β-catenin drastically decreased the lipogenic factor SREBP-1c [49].

Similar to galantamine, the effect of vildagliptin on lipid profile may be indebted to the activation of insulin/Wnt signaling pathways, as well as to the maintenance of persistent levels of active GLP-1 and GIP. The two incretin hormones reduce fasting lipolysis in adipose tissue and lower stored TGs in muscle, liver and pancreas [56]. Previous studies also reported a decrease in serum TC, TGs, and LDL-C in patients treated by vildagliptin [57]. Once again combining both drugs showed better impact on lipid profile in the tested organs, highlighting, thus, the positive interaction between both drugs.

Oxidative stress is one arm of the T2DM pathogenesis, which can directly or indirectly disturb functions of cellular macromolecules and activate cellular stress-sensitive signaling pathways [58]. Among these, is the nuclear transcription factor Nrf2 that controls the expression and the induction of a battery of defensive genes encoding detoxifying enzymes and antioxidants, by which mammalian cells can sense and adapt to oxidative stresses [59]. The present diabetic model depleted Nrf2 in both liver and muscle, a finding that goes in line with Pi et al. [58]. The authors stated that on the molecular level, Nrf2-mediated antioxidant response plays a paradoxical role in insulin secretion; under low levels of harmful stimuli, β-cells can adapt adequately by activating the Nrf2 system to minimize oxidative damage-related impairment of insulin secretion. However, under chronic exposure conditions, the adaptive endogenous antioxidant capacity is curtailed and can interfere with glucose-dependent endogenous reactive oxygen species (ROS) signaling. Consequently, a detrimental decrease in glucose stimulated insulin secretion occurs, as in the current model, which in turn confounds the Nrf2 system [58]. The imbalanced redox system further entailed the elevation of lipid peroxides and the decay in TAC level. In the present work, both drugs conveyed their antioxidant potentials by enhancing the TAC and the transcriptional activation of Nrf2 along with reducing the lipid peroxide level. Previously, Melo *et al*. [60] proved the galantamine beneficial antioxidant effect through the reduction of lipid peroxidation and the replenishment of glutathione stores. The antioxidant effect may be related, at least in part, to the inhibition of AChE, where increased oxidative load parallels the activated AChE, as reported in several studies [61, 62]. Vildagliptin, as well, prevents stress-induced destruction of pancreatic β-cells [63], kidney [19] and brain [64] *via* reducing the levels of lipid peroxidation, and enhancement of the antioxidant defense system. The antioxidant effect of vildagliptin can clarify, in part, the decreased AChE in brain, where it was reported previously that some antioxidants, such as curcumin [29] and vitamin E [65] prevented the cognitive deficits induced by the diabetic state through the inhibition of AChE.

In T2DM, the altered glucose homeostasis is accompanied by a constellation of consequences; including mitochondrial dysfunction, which represents a crucial source of increased ROS and apoptosis. In the present work, the n5-STZ model increased the tested apoptotic biomarkers, *viz*., cytochrome c and caspase-3, in liver and muscle. These results are in line with an earlier study, where the co-authors stated that in hepatocytes, impaired mitochondria amplifies the apoptotic signal pathway resulting in the release of several proapoptotic proteins into the cytosol, including cytochrome c, which in turn activates the downstream effector caspase-3 [66]. Additionally, apoptosis develops in myocytes *via* lipotoxicity induced by high saturated fatty acids documented herein and in a previous study [67]. Treatment with galantamine reduced both apoptotic markers, possibly through reducing fatty acids and/or inhibiting AChE, where ample of evidence point to the involvement of AChE in apoptosis, especially that induced by selective stressors as hyperglycemia [30]. Though it was less pronounced than that of galantamine, vildagliptin proved its antiapoptotic capacity, possibly by inhibiting DPP-4. This effect is in line with recent findings in Parkinson's disease model [64]. In 2014, Williams *et al*. [68] reported that the DPP-4 activity correlates with measures of hepatocyte apoptosis and fibrosis in T2DM and/or obesity. Once again, combining both drugs mediated the superior inhibition.

Overproduction of ROS/free radicals and hyperglycemia orchestrate an upsurge of a plethora of cytokines, with the adipose tissue depots being the central node for driving local and systemic inflammation along with I/R [69]. In the current study, galantamine induced a shift towards an anti-inflammatory phenotype, evidenced by the increase of adiponectin, and the decrease of the transcription factor NF-κB, the signature cytokine TNF-α [70], and visfatin, which were elevated in the diabetic animals. The ability of galantamine to abate TNF-α level in non-diabetic models [5, 71] may be attributed to the stimulation of the cholinergic anti-inflammatory pathway *via* the activation of α7nAChR [37, 72, 73] and/or elevation of adiponectin [5]. Although no direct data are available to correlate galantamine with NF-κB, yet inhibition of TNF-α and visfatin can be one possible pathway for the reduction of NF-κB [74] and *vice versa* [75, 76]. NF-κB was reported to trigger a signaling cascade, leading to a vicious cycle of oxidative stress and inflammation, to disrupt systemic insulin sensitivity and impair glucose homeostasis [77]. Moreover, in this work, low TNF-α level is responsible, at least in part, for the adjustment of fatty changes and the decrease of ALT and AST [78]. Vildagliptin also proved its effect against the inflammatory milieu, by increasing adiponectin and lowering the inflammatory markers tested, effects that are recently documented in a Parkinson's disease model [64] and in diabetic patients [79, 80].

In conclusion, our results demonstrate the capability of galantamine to alleviate T2DM and other components of the metabolic syndrome. These effects may be attributed to activation of the insulin and Wnt/β-catenin signaling pathways, beside its AChE inhibitory effect, anti-inflammatory action and antioxidant/antiapoptotic characters. The study points to the useful effect of galantamine as an add-on drug with antidiabetics, a common trend in the time being for the management of T2DM.

## Supporting Information

S1 Checklist “The ARRIVE Guidelines Checklist” for reporting animal data in this manuscript. (DOCX)
