# Supplementary material for: Antidiabetic Effect of Galantamine: Novel Effect for a Known Centrally Acting Drug
Source: PLoS One. 2015 Aug 11;10(8):e0134648. doi: 10.1371/journal.pone.0134648 (PMC4532414; doi:10.1371/journal.pone.0134648)

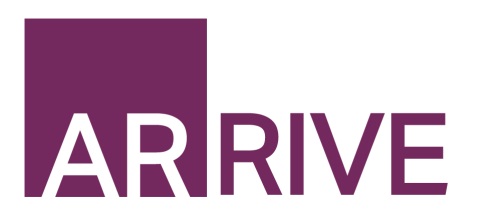


The ARRIVE Guidelines Checklist

Animal Research: Reporting In Vivo Experiments

Carol Kilkenny^1^, William J Browne^2^, Innes C Cuthill^3^, Michael Emerson^4^ and Douglas G Altman^5^

*^1^The National Centre for the Replacement, Refinement and Reduction of Animals in Research, London, UK, ^2^School of Veterinary Science, University of Bristol, Bristol, UK, ^3^School of Biological Sciences, University of Bristol, Bristol, UK, ^4^National Heart and Lung Institute, Imperial College London, UK, ^5^Centre for Statistics in Medicine, University of Oxford, Oxford, UK.*

|  | | ITEM | RECOMMENDATION | Section/ Paragraph |
| --- | --- | --- | --- | --- |
| 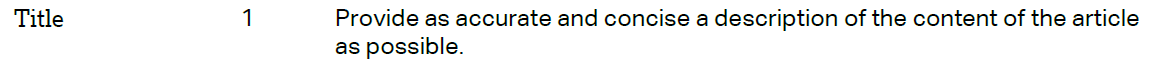 | | | Title |  |
| 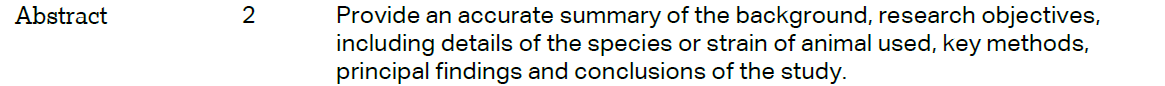 | | | Abstract |  |
| INTRODUCTION | | |  |  |
| 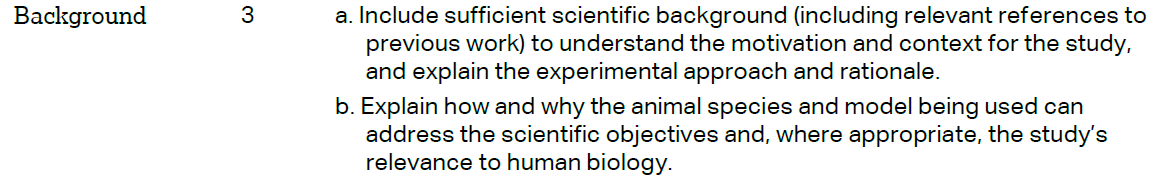 | | | Paragraphs 1 and 3-6  Materials & Methods, paragraph 1  Discussion paragraph 2 |  |
| 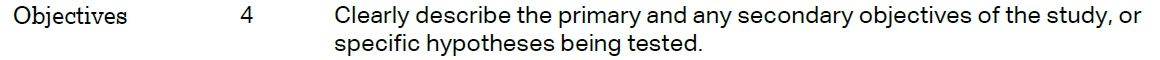 | | | Paragraph 6 |  |
| METHODS | | |  |  |
| 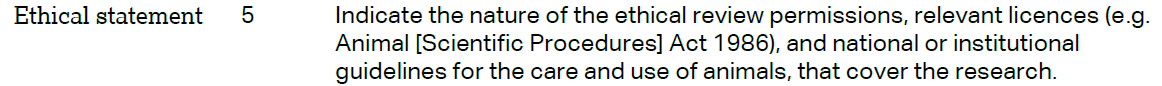 | | | Paragraph 2 |  |
| 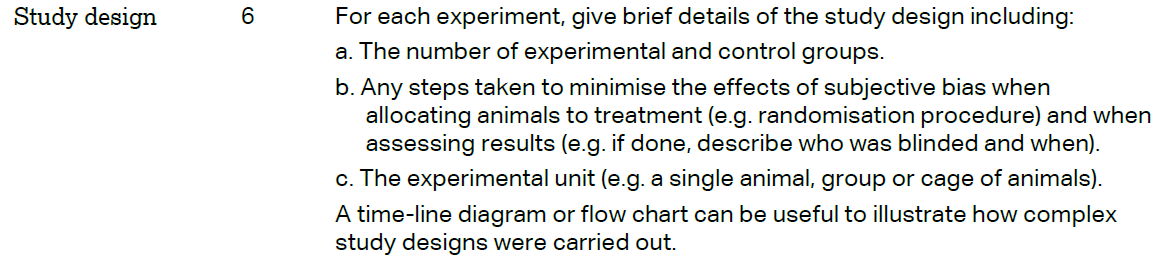 | | | Paragraphs 1, 3 |  |
| 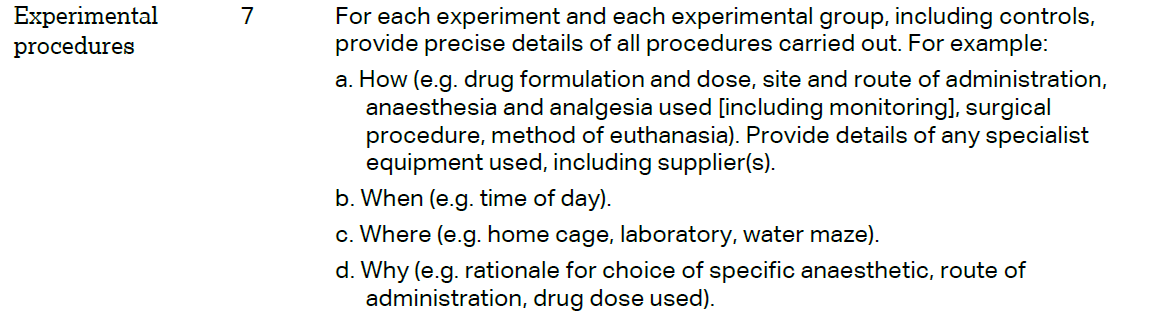 | | | Paragraphs 1, 3, 5 |  |
| 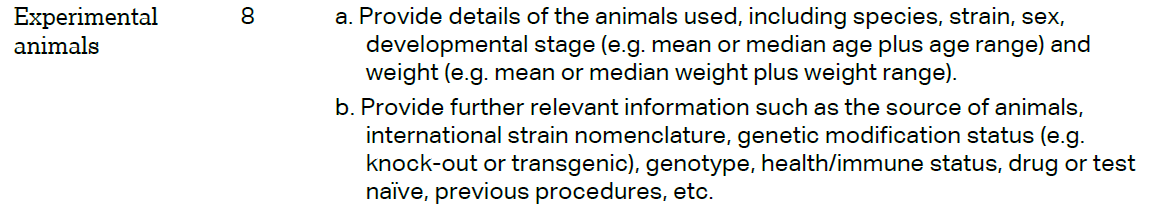 | | | Paragraph 1 |  |

The ARRIVE guidelines. Originally published in *PLoS Biology*, June 2010^1^

| 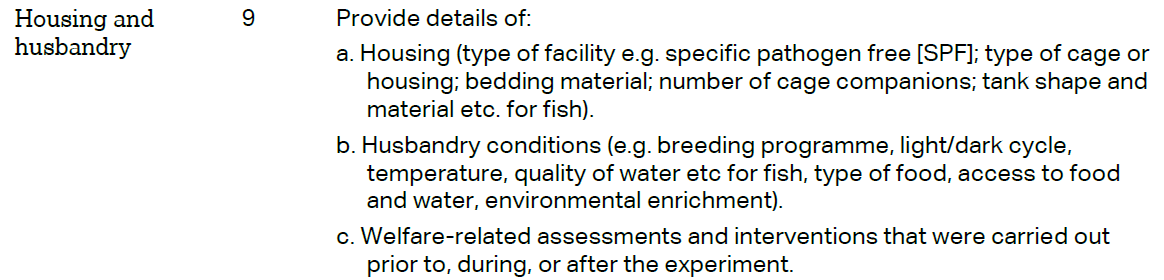 | Paragraph 1 | |
| --- | --- | --- |
| 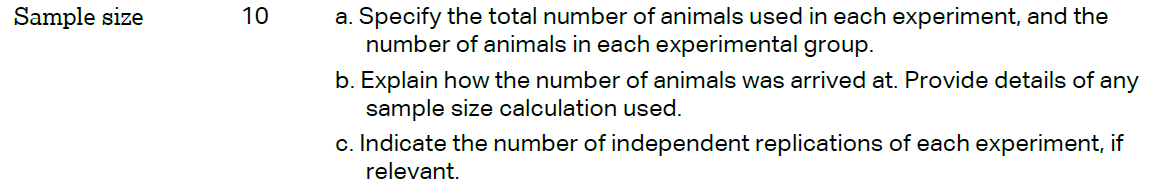 | Paragraphs 1, 3 | |
| 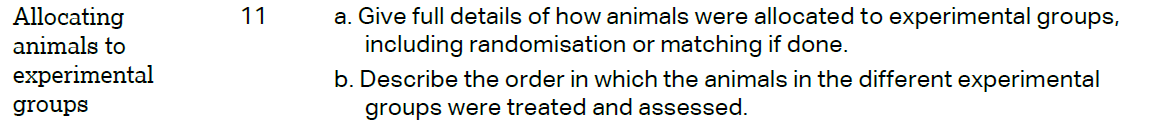 | Paragraphs1, 3 | |
| 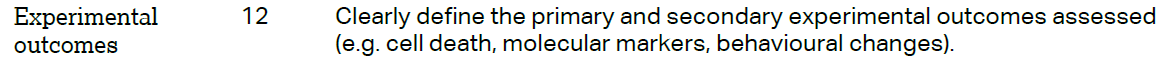 | Paragraphs4-14 | |
| 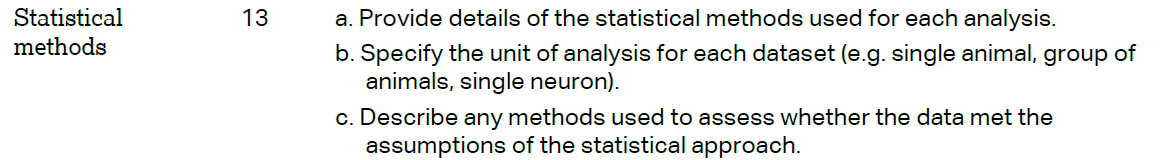 | Paragraph 15 | |
| RESULTS |  | |
| 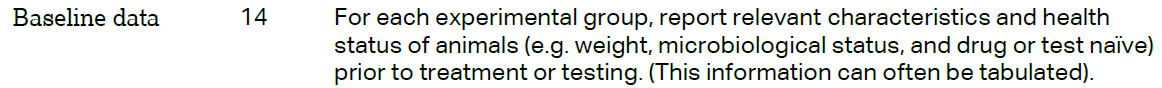 | Paragraph 1  Paragraph 1, table 1 | |
| 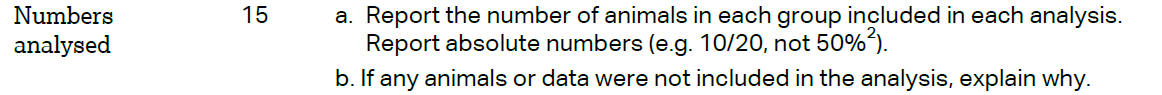 | Materials & Methods paragraph 1 | |
| 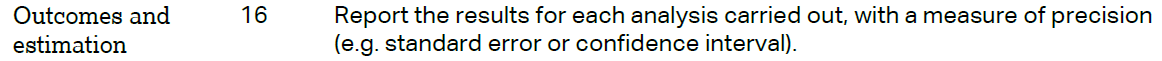 | Paragraphs 1-12  Figures 1-12  Tables 1, 2 | |
| 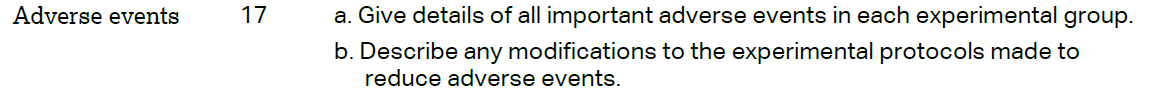 | No adverse events | |
| DISCUSSION |  | |
| 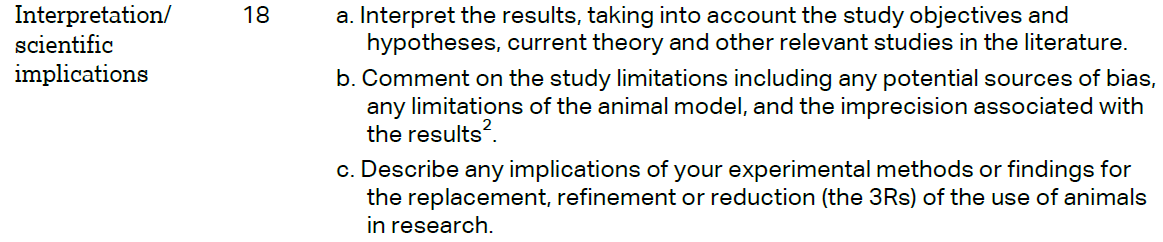 | Throughout | |
| 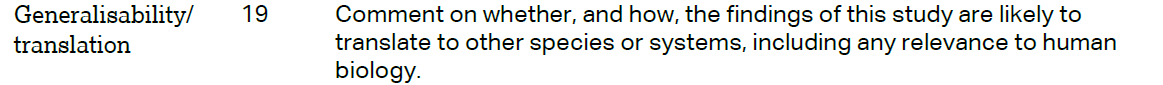 | Paragraph 12 | |
| 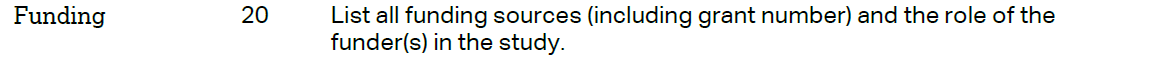 | | No funding agencies |


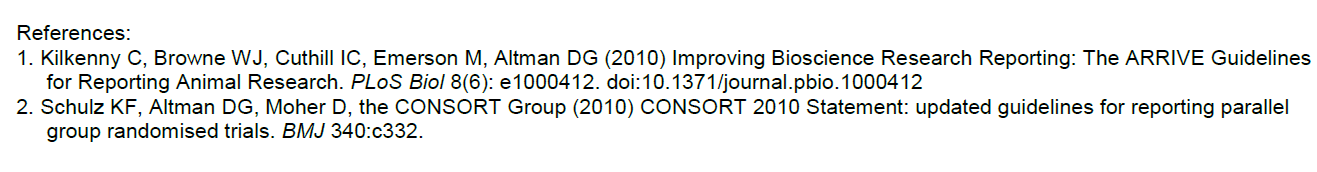

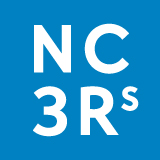

Supplement: S1 Checklist — (DOCX) [file pone.0134648.s001.docx]
